# Structures of LIG1 uncover the mechanism of sugar discrimination against 5′-RNA-DNA junctions during ribonucleotide excision repair

**DOI:** 10.1016/j.jbc.2024.107688

**Published:** 2024-08-17

**Authors:** Kanal Elamparithi Balu, Qun Tang, Danah Almohdar, Jacob Ratcliffe, Mustafa Kalaycioğlu, Melike Çağlayan

**Affiliations:** Department of Biochemistry and Molecular Biology, University of Florida, Gainesville, Florida, USA

**Keywords:** genome stability, DNA repair, DNA replication, ribonucleotide excision repair, DNA ligase 1, nick sealing, DNA ligation, ribonucleotide, sugar discrimination

## Abstract

Ribonucleotides in DNA cause several types of genome instability and can be removed by ribonucleotide excision repair (RER) that is finalized by DNA ligase 1 (LIG1). However, the mechanism by which LIG1 discriminates the RER intermediate containing a 5′-RNA–DNA lesion generated by RNase H2-mediated cleavage of ribonucleotides at atomic resolution remains unknown. Here, we determine X-ray structures of LIG1/5′-rG:C at the initial step of ligation where AMP is bound to the active site of the ligase and uncover a large conformational change downstream the nick resulting in a shift at Arg(R)871 residue in the Adenylation domain of the ligase. Furthermore, we demonstrate a diminished ligation of the nick DNA substrate with a 5′-ribonucleotide in comparison to an efficient end joining of the nick substrate with a 3′-ribonucleotide by LIG1. Finally, our results demonstrate that mutations at the active site residues of the ligase and LIG1 disease-associated variants significantly impact the ligation efficiency of RNA–DNA heteroduplexes harboring “wrong” sugar at 3′- or 5′-end of nick. Collectively, our findings provide a novel atomic insight into proficient sugar discrimination by LIG1 during the processing of the most abundant form of DNA damage in cells, genomic ribonucleotides, during the initial step of the RER pathway.

Intracellular concentrations of ribonucleotide triphosphates (rNTPs) exceed those of deoxyribonucleotide triphosphates (dNTPs) and DNA polymerases extensively use rNTPs instead of dNTPs; only a single oxygen atom being different between the two nucleotide substrates, for genomic incorporation (1). Ribonucleotides are indeed the most frequently incorporated non-canonical nucleotides in duplex DNA, exceeding a combined total number of abasic sites and oxidative DNA damage (2). Approximately 13,000 and 1,000,000 ribonucleotides are embedded into the genomes of yeast and mouse embryonic fibroblast cells, respectively (3, 4, 5). Most DNA polymerases possess a highly conserved “steric gate” to prevent rNTP incorporation, while others utilize a protein backbone segment (*i.e..*, X-family polymerases β and λ) that governs ribonucleotide exclusion (6, 7, 8, 9, 10, 11, 12, 13, 14). Despite this sugar discrimination, replicative and repair DNA polymerases incorporate rNTPs at a remarkably high rate (15). Particularly, rNTPs are incorporated into the genome during replication as observed for yeast replicative DNA polymerases that insert one ribonucleotide for every thousands of deoxyribonucleotides (13). Furthermore, an imperfect nucleotide selectivity of replicative DNA polymerases α, δ, and ε, and inefficient removal of ribonucleotides through their 3′–5′ exonuclease proofreading activities lead to aberrant ribonucleotide incorporation more frequently than those of mismatches during DNA replication (16, 17). Genomic ribonucleotides can cause several types of genome instability. The presence of ribonucleoside monophosphates (rNMPs) in the context of DNA template directly affects the processivity of DNA polymerization during replication, and induces replication stress and DNA damage signaling from yeast to humans (2, 18, 19). Furthermore, ribonucleotides embedded into genomic DNA can confound the structural and chemical integrity of duplex DNA, increase its susceptibility to endogenous or exogenous damage, and lead to mutagenesis, aberrant recombination, protein–DNA crosslinks, double-strand breaks, and chromosome alterations (1, 2, 16, 17).

Ribonucleotide excision repair (RER) is the primary mechanism of rNMP removal from genomic DNA and involves a coordinated function of RER enzymes in a multistep process (20). In the initial step of the RER pathway, RNase H2 recognizes an rNMP in the context of duplex DNA and incises the DNA backbone on the 5′-side of the ribonucleotide generating DNA containing a 3′-hydroxy (3′-OH) group and a 5′-phosphate (PO_4_) end to allow its subsequent processing (21, 22, 23). DNA polymerase (pol) δ then performs a strand displacement DNA synthesis, thereby creating a flap structure harboring the rNMP (24). This flap is subsequently removed by Flap endonuclease 1 (FEN1) or exonuclease (Exo) and the remaining single-stranded nick is sealed by DNA ligase 1 (LIG1) at the final step of the RER pathway (25). Mutations in RNase H2 are associated with an autosomal recessive disorder, Aicardi–Goutières syndrome, and patients exhibit progressive microencephaly and neurological defects (26). Embryonic lethality at the gastrulation stage of development resulting from the complete disruption of RNase H2 has been reported in homozygous null mice (27). In the absence of functional RNase H2, embedded ribonucleotides are repaired by an alternate pathway involving DNA topoisomerase that relaxes negatively supercoiled DNA by transiently cleaving and re-ligating one or both strands of DNA (28).

During RER pathway, the RNA–DNA junctions can be formed in cells when RNase H2 initiates the processing of embedded ribonucleotides (29). This RER intermediate is a nick containing 3′-OH and a ribonucleotide at the 5′-end, which competes with the regular substrate of human DNA ligase that requires canonical 3′-OH and 5′-PO_4_ termini to be efficiently sealed. LIG1 attempts to ligate the RER intermediate following RNase H2-mediated cleavage and fails on the RNA–DNA junctions resulting in the formation of abortive ligation products with a 5′-adenylated-ribonucleotide, 5′-AMP-RNA (30). Aprataxin (APTX) removes a 5′-AMP from these abortive ligation products and mutations in *APTX* cause an autosomal recessive neurodegenerative disease, Ataxia with Oculomotor Apraxia Type 1 (31, 32). Furthermore, this mechanism promotes cellular survival and prevents S phase checkpoint activation in budding yeast (30). LIG1 is involved in earlier steps of the RER pathway; however, how the active site of the ligase surveils RNA–DNA junctions at atomic resolution and structural insight into sugar discrimination against a 5′-ribonucleotide at nick DNA by LIG1 are largely unexplored.

In addition to RER, LIG1 plays a crucial role in Okazaki fragment maturation during DNA replication and completes DNA repair pathways (33, 34). To maintain the integrity of double-helix structure during DNA replication and repair at the final step, LIG1 ensures high fidelity during a three-step ligation reaction (Scheme S1): the formation of DNA ligase–adenylate intermediate (LIG–AMP) in step 1, subsequent transfer of an AMP moiety to the 5′-PO_4_ end of the nick (DNA–AMP) in step 2, and finally, a phosphodiester bond formation coupled to AMP release in step 3 (35). In our previous studies on LIG1 structures (36, 37, 38), we have uncovered how the active site of the ligase engages with unusual ends during this three-step ligation reaction. For example, we have reported the structures of LIG1/nick complexes containing A:C and G:T mismatches that mimic the repair intermediates after incorporation of mismatches by DNA polymerases, and demonstrated the strategies that the active site of the ligase employs to deter (step 1, A:C) or favor (step 2, G:T) mispairs owing to polymerase errors (36). Furthermore, our recent study on LIG1 structures in complex with oxidatively damaged DNA and RNA (8oxodG and 8oxorG) has demonstrated how the ligase engages with mutagenic repair intermediates through movements at the nucleotides around nick site depending on dual coding potential of 8oxodGTP(*anti*):C(*anti*) and 8oxodGTP(*syn*):A(*anti*) that forms non-mutagenic Watson–Crick and mutagenic Hoogsteen base pairing, respectively (37). In addition to these LIG1 structures, we have recently reported the mechanism, by which LIG1 surveils “wrong” sugar at a nick upon incorporation of rNTPs by repair and replication polymerases (38). We have reported in the LIG1/DNA–RNA heteroduplex structures that the ligase lacks sugar discrimination against a single ribonucleotide at the 3′-end of nick DNA (38). We have revealed that the active site of the ligase utilizes a network of LIG1/DNA interactions through Asp570 and Arg871 side chains with 2′-OH of the ribose during the final phosphodiester bond formation step of the ligation reaction, which overall enables ligase to efficiently seal a nick containing a 3′-ribonucleotide at the final step of DNA repair and replication (38).

In the present study, using X-ray crystallography, we aimed to elucidate the mechanism by which LIG1 discriminates against a single ribonucleotide at the 5′-end of nick DNA at atomic resolution. In addition, we comprehensively investigated the ligation efficiency of LIG1 (wild-type, active site mutants, and disease-associated variants) for the nick DNA substrates containing a ribonucleotide at the 3′- or 5′-end *in vitro*. Our LIG1 structures revealed proficient sugar discrimination against 5′-RNA–DNA junctions that could be formed after RNase H2-dependent excision during the RER pathway. We captured the LIG1/5′-rG:C structures at the initial step of ligation reaction where the active site (K568) of the ligase stays adenylated (LIG1–AMP). We demonstrated a large conformational change downstream of the nick resulting in a narrowing of the minor groove interactions. The superimposition of LIG1/5′-rG:C structures with our previously resolved structures of LIG1 with canonical, mismatch, 3′-ribonucleotide that were captured at different steps of the ligation reaction revealed a shift at the Arg(R)871 residue in the Adenylation domain of the ligase. Furthermore, we demonstrated diminished nick sealing in the presence of 5′-ribonucleotide compared to an efficient ligation of nick DNA containing 3′-ribonucleotide by LIG1. We also showed the impact of mutations at the active site residues (F635A, F872A, and R738A) of LIG1 on the ligation of nick DNA with 5′-rG:C. Finally, our results revealed a notable difference in the end-joining abilities of LIG1 deficiency disease-associated variants P529L, R641L, and R771W in the presence of nick DNA substrates containing 3′-rG:C *versus* 5′-rG:C. Overall, our findings provide a novel atomic insight into sugar discrimination by LIG1 against 5′-RNA–DNA junctions and reveal how the active site of the ligase deters sealing of nicks with 5′-ribonucleotide during RER.

## Results

### LIG1 discriminates against RNase H2-dependent excision repair lesions containing a ribonucleotide at the 5′-end of nick DNA

**Figure 1:**
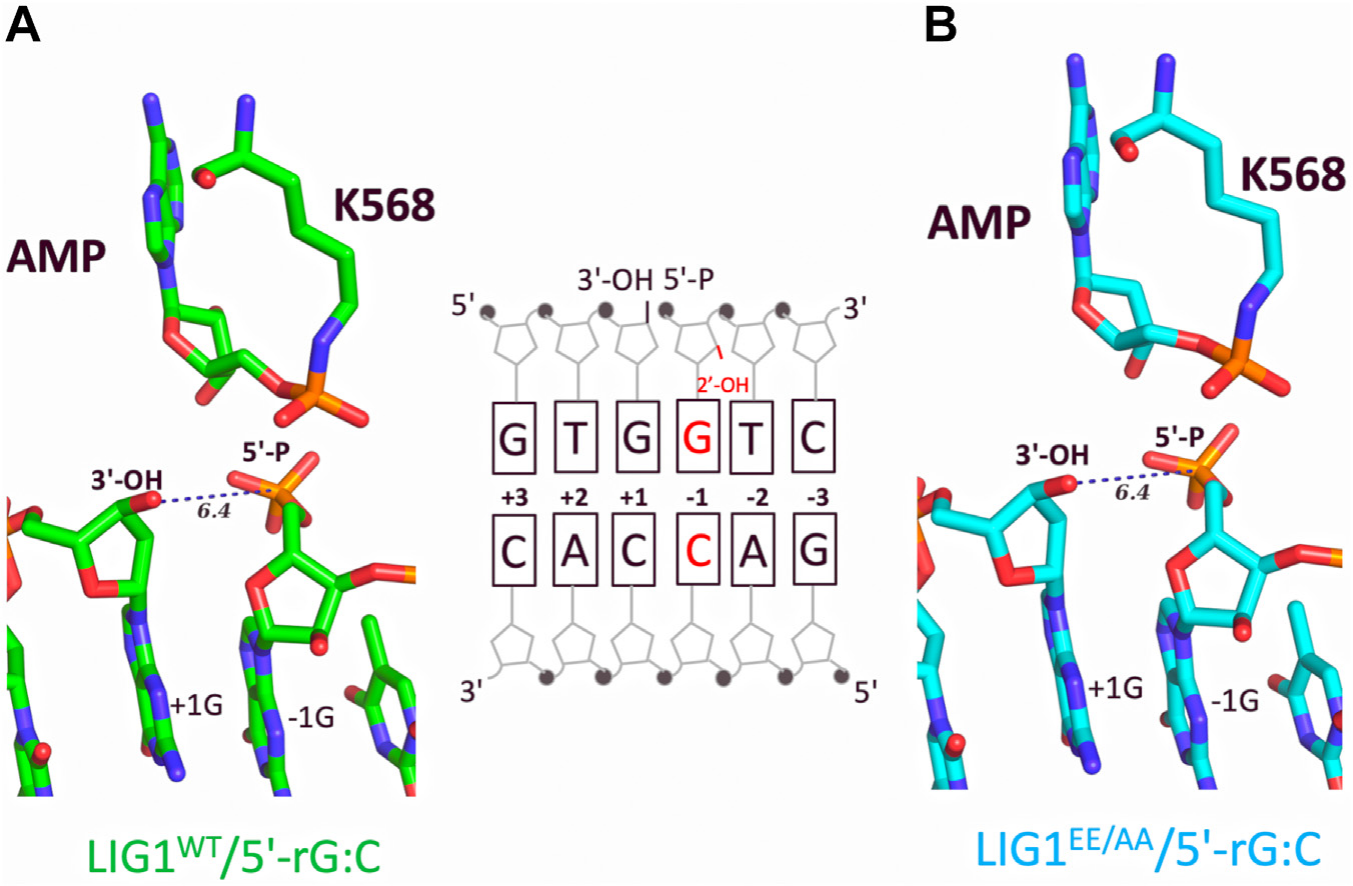
**Structures of LIG1 bound RNA/DNA heteroduplexes containing 5′-ribonucleotide at the nick.***A* and *B*, Structures of LIG1^WT^ and LIG1^EE/AA^ in complex with nick DNA containing 5′-rG:C demonstrate step 1 of the ligation reaction where AMP is bound to the ligase active site (K568). Schematic view of the nick DNA shows the sequence of 3′- and 5′-terminus of the nick DNA substrate used in crystalization.

In our crystallization studies, we used LIG1 wild-type (LIG1^WT^) and EE/AA mutant (LIG1^EE/AA^) that harbors E346A and E592A mutations, resulting in the ablation of the high-fidelity site (referred to as Mg^HiFi^ site). This mutant has been previously utilized to crystalize LIG1 with nick DNA containing non-canonical ends owing to mismatched or oxidative damage (36, 37, 38, 39, 40). We solved the structures of LIG1^WT^ and LIG1^EE/AA^ in complex with DNA/RNA junction harboring a 5′-ribonucleotide at the nick (Fig. 1 and Table 1). We captured LIG1^WT^ (Fig. 1*A*) and LIG1^EE/AA^ structures (Fig. 1*B*) at step 1 of ligation reaction when an AMP moiety is covalently bound to the active site residue K568 of the ligase (Fig. S1). The root mean square deviation (RMSD) was 0.190 Å, demonstrating that both structures share similar global conformation (Table S1).

**Table 1:** X-ray data collection and refinement statistics

	LIG1^WT^ 5′-rG:C 9BS3	LIG1^EE/AA^ 5′-rG:C 9BS4
Data collection		
Space group	P2_1_	P2_1_
Cell dimensions		
*a*, *b*, *c* (Å)	71.3 117.2 104.0	71.3 117.2 101.5
α, β, γ (°)	90.0 98.8 90.0	90.0 96.7 90.0
Resolution (Å)	25–2.69 (2.75–2.69)	20–2.4 (2.44–2.4)
*R*_pim_	0.074 (0.313)	0.066 (0.437)
*I*/σ (*I)*	13.0 (1.8)	17.0 (1.6)
*CC*_*1/2*_	0.980 (0.811)	0.981 (0.599)
*CC∗*	0.995 (0.946)	0.995 (0.865)
Completeness (%)	97.6 (99.3)	99.9 (99.8)
Redundancy	6.2 (6.3)	6.7 (6.1)
Refinement		
Resolution (Å)	25–2.69	20–2.40
No. reflections	45,425	59,693
*R*_work_/*R*_free_	20.8/24.9	19.0/23.7
Non-H atoms	10,237	10,461
Protein	8582	8572
DNA/RNA	1471	1468
AMP	44	44
H_2_O	140	377
Average B-factors (Å^2^)	50.5	43.1
Protein	50.9	42.8
DNA	48.7	46.1
AMP	43.4	34.6
H_2_O	44.7	41.2
R.M.S.D		
Bond lengths (Å)	0.003	0.004
Bond angles (°)	0.557	0.596

**Figure 2:**
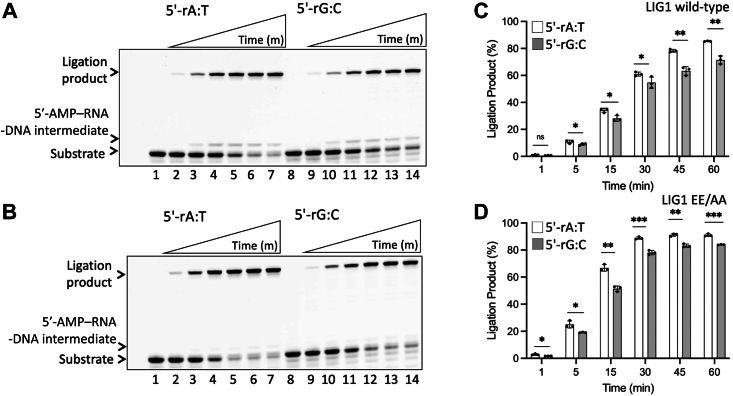
**Ligation of thenick DNA substrate with a ribonucleotide at the 5′-end by LIG1.***A* and *B*, lanes 1 and 8 are the negative enzyme controls of the nick DNA substrates with 5′-rA:T and 5′-rG:C, respectively. Lanes 2 to 7 and 9 to 14 are the ligation products by LIG1 wild-type (*A*) and EE/AA mutant (*B*) in the presence of 5′-rA:T and 5′-rG:C, respectively, and correspond to time points of 1, 5, 15, 30, 45, and 60 min. *C* and *D*, graphs show the time-dependent change in the amount of ligation products and the data represent the average from three independent experiments ± SD; ns *p* > 0.05; ∗*p* < 0.05; ∗∗*p* < 0.01; ∗∗∗*p* < 0.001; ∗∗∗∗*p* < 0.0001.

We then tested the ligation efficiency of LIG1 using the nick DNA substrates with 5′-rA:T and 5′-rG:C (Scheme S2). The results demonstrated ligation of nick DNA with a 5′-ribonucleotide particularly for long incubation times, *i.e*., up to 60 min (Fig. 2, *A* and *B*). A time-dependent increase in the amount of ligation products was similar by both LIG1 proteins (Figs. 2, *C*, *D* and S2). The ligase failure products with 5′-AMP–RNA-DNA also accumulated along with the ligation products in the presence of 5′-rA:T and 5′-rG:C (Fig. S3, *A* and *B*). In the control experiments, we observed an efficient ligation of nick DNA containing a canonical end (3′-dG:C) by LIG1 wild-type and EE/AA mutant (Fig. S4).

### Structures of LIG1/RNA–DNA heteroduplex with a 5′-ribonucleotide demonstrate a shift at R738 residue

**Figure 3:**
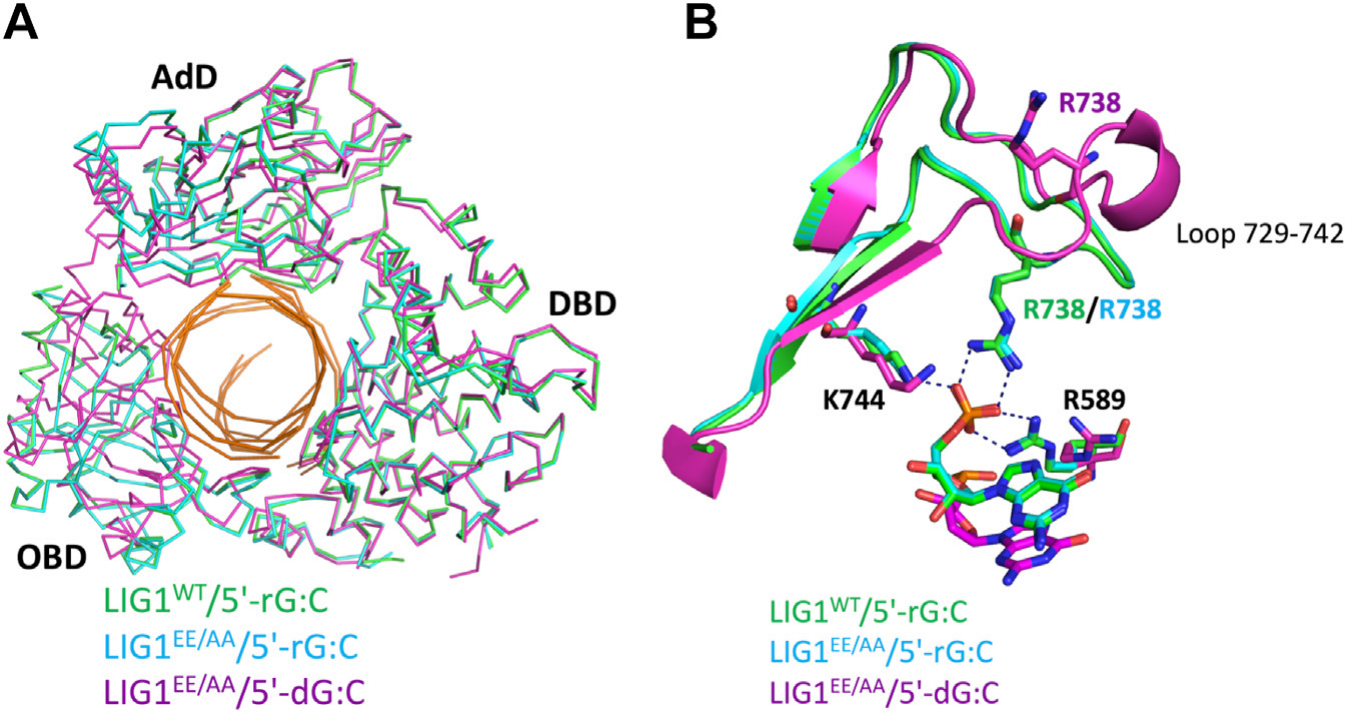
**Overlays of LIG1 structures in complex with nick DNA containing canonical *versus* 5′-ribonucleotide-containing ends.***A* and *B*, the superimposition of LIG1^EE/AA^/5′-rG:C (step 1) and LIG1^WT^/5′-rG:C (step 1) structures with LIG1/5′-dG:C (step 2) demonstrate that the AdD, OBD, and DBD domains of the ligase show the same global conformation (*A*) and a shift in the loop (amino acids of 729–742) including R738 residue in the AdD domain that interacts with 5′-PO_4_ end of nick in the structure of LIG1/5′-rG:C (*B*).

To comprehensively understand the mechanism by which LIG1 discriminates a 5′-ribonucleotide, we overlayed LIG1 structures with 5′-rG:C onto our previously solved structure of LIG1 in complex with nick DNA containing canonical 5′-dG:C (Fig. 3). The RMSD was 1.027 Å. The superimposition of LIG1^WT^/5′-rG:C and LIG1^EE/AA^/5′-rG:C structures showed that the oligonucleotide-binding domain (OBD), adenylation domain (AdD), and DNA-binding domain (DBD) of the ligase encircle the nick, and the DBD domain only share a similar conformation (Fig. 3*A*). This superimposition also demonstrated a shift of ∼15 Å in the AdD domain of ligase, particularly in the region corresponding to amino acids between 729 to 742 including the active site residue, Arg(R)738, of the ligase toward 5′-PO_4_, which was observed only in the structure of LIG1/5′-rG:C (Fig. 3*B*).

**Figure 4:**
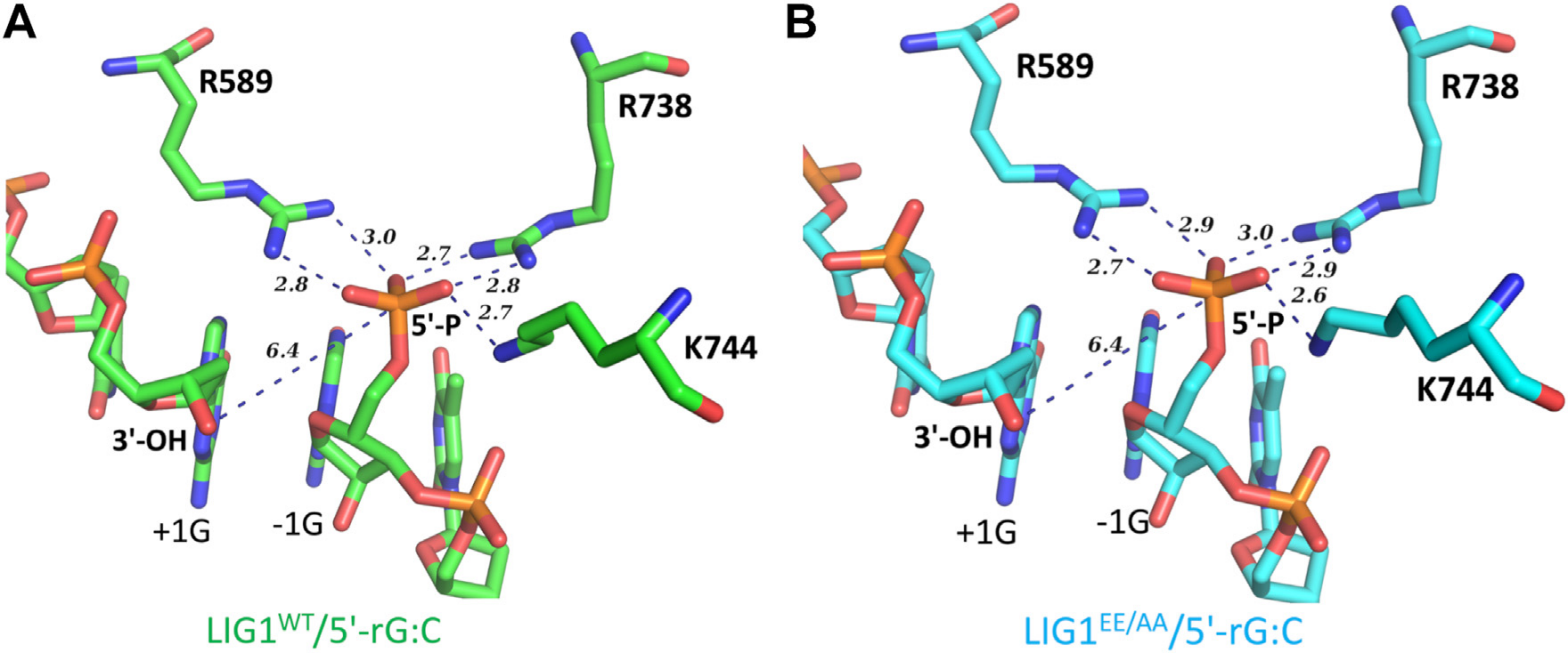
**LIG1/5′-rG:C structures demonstrate an interaction network of the ligase active site residues.***A* and *B,* structures of LIG1^WT^/5′-rG:C (*A*) and LIG1^EE/AA^/5′-rG:C (*B*) show the interaction network and distances between the ligase active site residues R589, R738, K744 and 5′-PO_4_ end of nick.

Furthermore, we observed, in both structures of LIG1/5′-rG:C, that the active site residues Arg(R)738, Lys(K)744, and Arg(R)589 form salt bridges with 5′-PO_4_ (Fig. 4) and the shift at R738 form a putative hydrogen bond with 5′-PO_4_ as observed from the superimposition of LIG1/5′-rG:C structures with our other previously reported structures of LIG1 in complex with nick DNA containing canonical, mismatch, or 3′-ribonucleotide (Fig. S5). We suggest that this shift at R738 could deter AMP transfer from the active site (K568) of the ligase to the 5′-end of nick DNA by increasing the energy barrier of adenylate transfer, which impedes with moving forward of ligation reaction from step 1 (LIG1–AMP) to step 2 (DNA–AMP).

**Figure 5:**
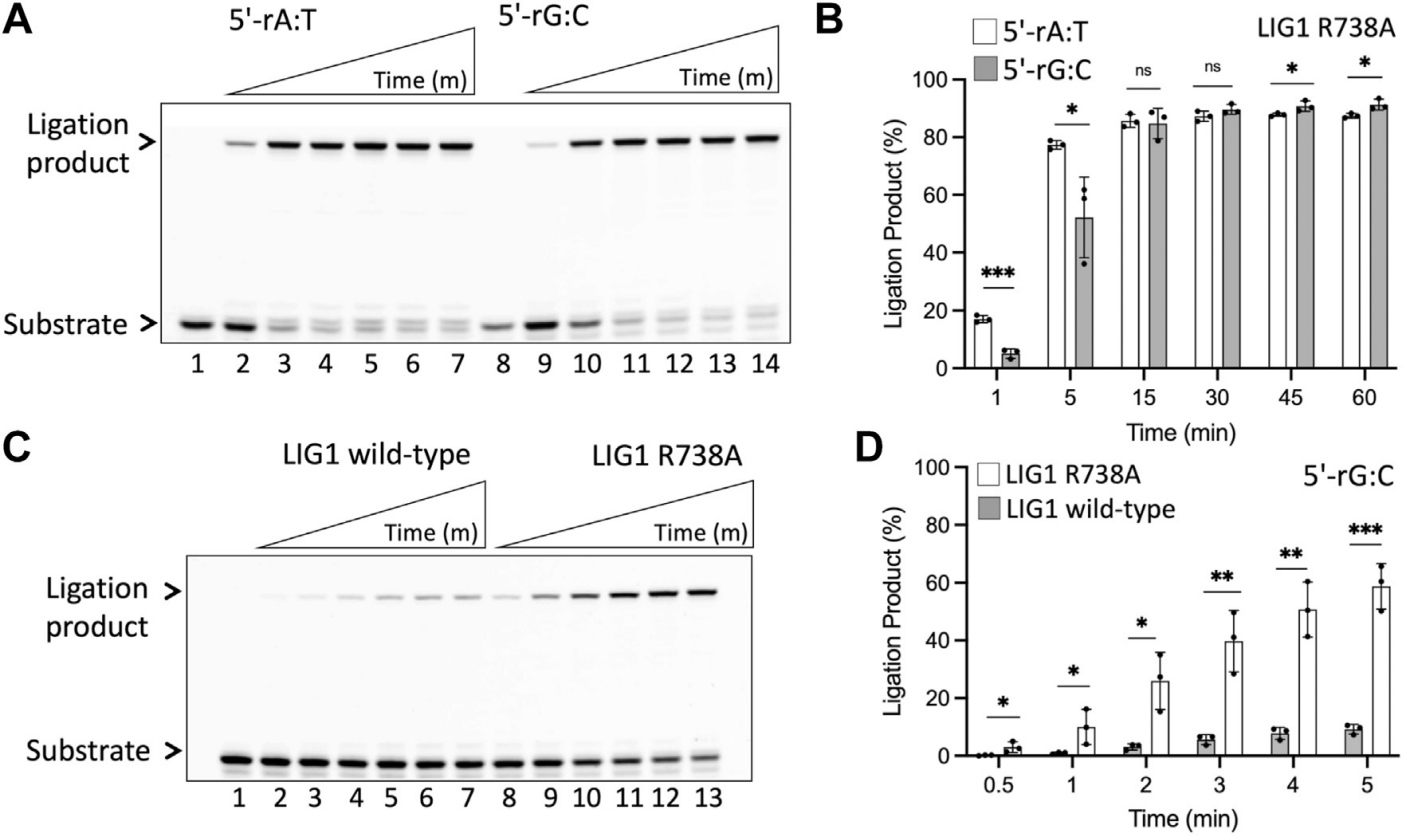
**Ligation of the nick DNA substrate with a ribonucleotide at the 5′-end by LIG1 R738A mutant.***A*, lanes 1 and 8 are the negative enzyme controls of the nick DNA substrates with 5′-rA:T and 5′-rG:C, respectively. Lanes 2 to 7 and 9 to 14 are the ligation products by LIG1 R738A mutant in the presence of 5′-rA:T and 5′-rG:C, respectively, and correspond to time points of 1, 5, 15, 30, 45, and 60 min. *C*, line 1 is the negative enzyme control of the nick DNA substrate with 5′-rG:C. Lanes 2 to 7 and 8 to 13 are the ligation products by LIG1 wild-type and R738A mutant, respectively, and correspond to time points of 0.5, 1, 2, 3, 4, and 5 min. *B and D*, graphs show the time-dependent change in the amount of ligation products and the data represent the average from three independent experiments ± SD; ns *p* > 0.05; ∗*p* < 0.05; ∗∗*p* < 0.01; ∗∗∗*p* < 0.001; ∗∗∗∗*p* < 0.0001.

To further understand the role of R738 residue showing a shift in the LIG1/5′-rG:C structure, we generated Arg(R) to Ala(A) substitution at 738 side chain position and performed ligation assay using LIG1 R738A mutant (Fig. 5). Our results demonstrated that R738A mutation led to efficient nick sealing by LIG1 in the presence of nick DNA substrates containing 5′-rA:T and 5′-rG:C (Fig. 5*A*) with a slight difference in the amount of ligation products (Fig. 5*B*). When we compared this efficiency with that of LIG1 wild-type for earlier time points of reaction (0.5–5 min), the results demonstrated that R738A mutation led to relatively efficient ligation of 5′-rG:C substrate (Fig. 5*C*). A comparison of ligation products showed ∼6-fold difference for the last time point (5 min) of the ligation reaction (Fig. 5*D*), suggesting a contribution of R738 to sugar discrimination against a 5′-ribonucleotide by LIG1. However, the ligation of nick substrate with a canonical end (3′-dG:C) by LIG1 R738A mutant was found to be slightly less than that of wild-type enzyme (Fig. S6).

### Structures of LIG1/DNA with 5′-ribonucleotide reveal a conformational change downstream of the nick

**Figure 6:**
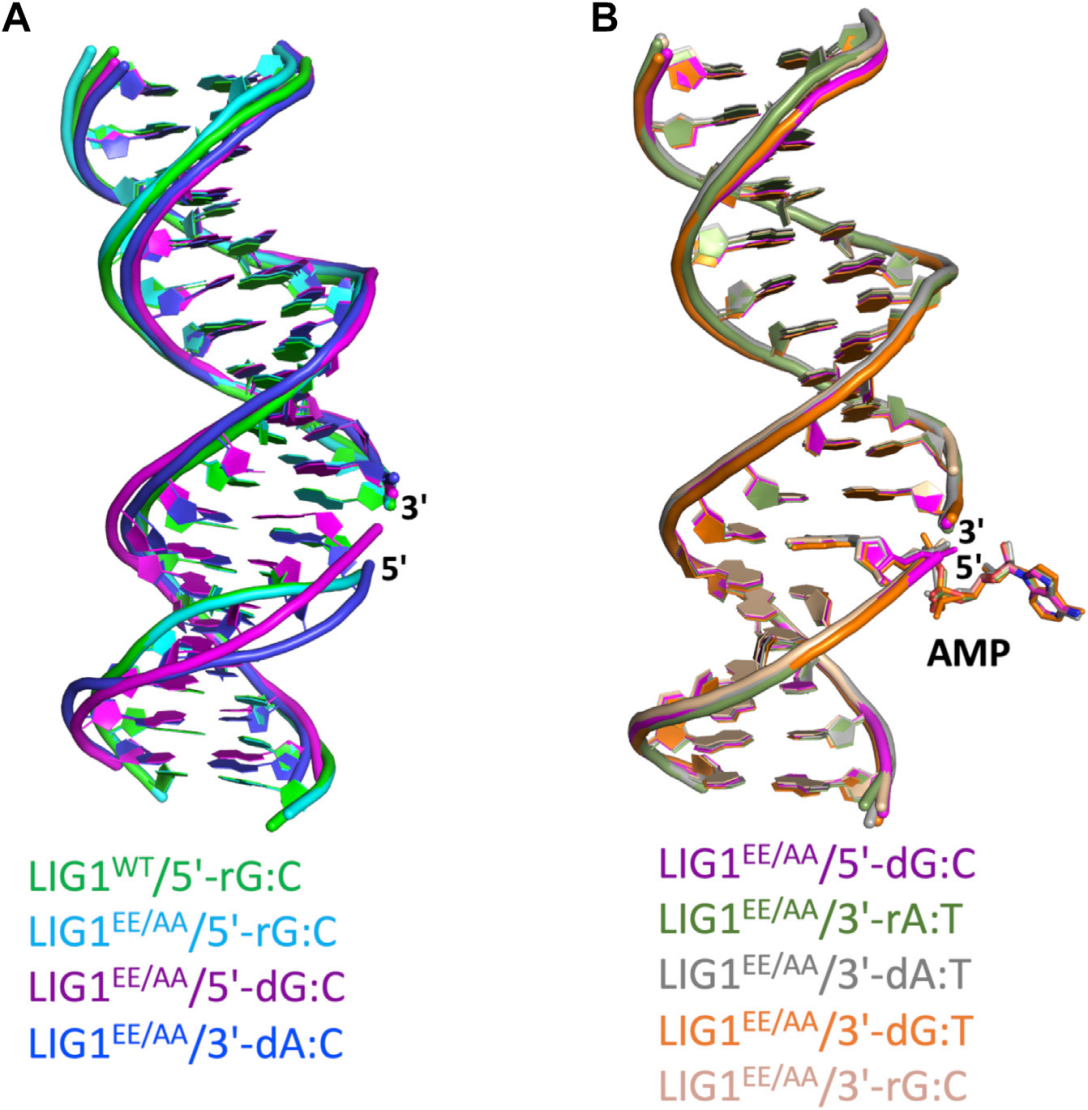
**Structure of LIG1/5′-rG:C reveals a conformational change in nick DNA.***A* and *B*, the superimposition of LIG1/5′-rG:C structures with previously solved structures of LIG1 in complex with nick DNA containing canonical, mismatch, and 3′-ribonucleotide shows a difference in the DNA conformation and demonstrates a conformational change in the downstream of the nick in the presence of 5′-ribonucleotide.

To further understand the mechanism of proficient sugar discrimination by LIG1 against 5′-ribonucleotide at the nick, we calculated the RMSD value that was >1 Å, indicating a large global conformational change, whereas the RMSD values have been found to be <1 Å for all other previously solved LIG1 structures (Table S1). To determine the cause of this global conformational change, we overlayed the nick DNA of LIG1/5′-rG:C structures (step 1) with our previously solved structures that were captured at steps 1, 2, or 3 of ligation reaction (Scheme S1), such as canonical 3′-dA:T, 3′-dG:C; mismatches 3′-dA:C, 3′-dG:T; and ribonucleotides 3′-rA:T, 3′-rG:C (Figs. 6 and S7). This superimposition demonstrated that the salt bridge observed in LIG1/5′-rG:C structures through the active site residues R738, K744, and R589 generate a conformational change downstream of the nick (Fig. 6*A*), whereas no change was observed in our previously reported LIG1 structures (Fig. 6*B*).

Amongst all LIG1 structures showing the catalytic core consisting of AdD, OBD, and DBD domains, we observed that the major difference was the rotation of 5′-PO_4_ downstream the nick DNA, which widened the major groove downstream the nick DNA and resulted in a narrowing of the minor groove (Fig. S8). The minor groove is the interaction site of the OBD, and the conformational change downstream could alter the interaction between the OBD and AdD, resulting in an increase in solvent accessible area at the minor groove where the OBD and AdD interact with the nick.

### Ligation efficiency of nick DNA containing a ribonucleotide at the 3′- or 5′-end by LIG1 wild-type and variants

**Figure 7:**
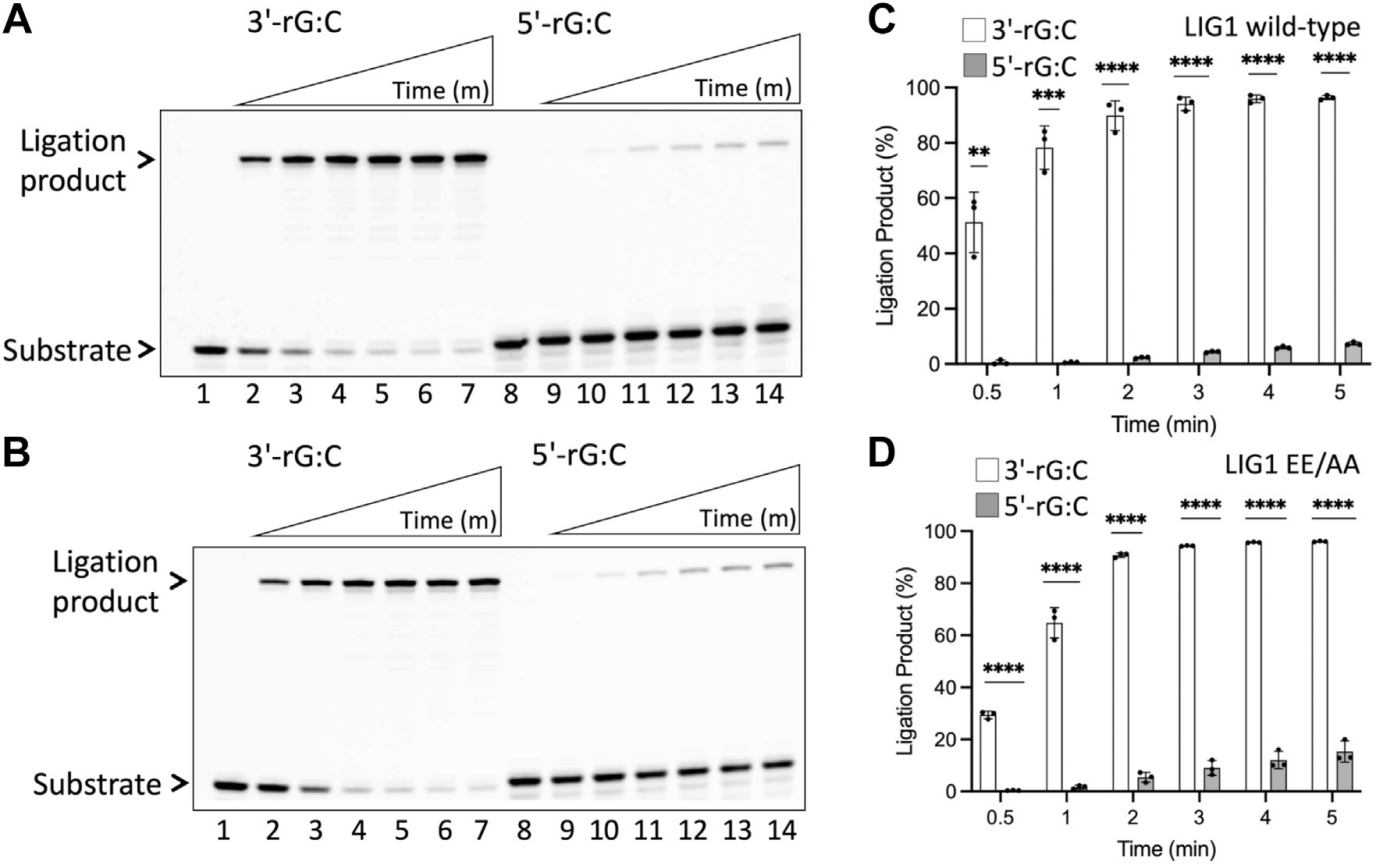
**Ligation of the nick DNA substrate with a ribonucleotide at the 3′- *versus* 5′-end by LIG1.***A* and *B*, Lanes 1 and 8 are the negative enzyme controls of the nick DNA substrates with 3′-rG:C and 5′-rG:C, respectively. Lanes 2 to 7 and 9 to 14 are the ligation products by LIG1 wild-type (*A*) and EE/AA mutant (*B*) in the presence of 3′-rG:C and 5′-rG:C, respectively, and correspond to time points of 0.5, 1, 2, 3, 4, and 5 min. *C* and *D*, graphs show the time-dependent change in the amount of ligation products and the data represent the average from three independent experiments ± SD; ns *p* > 0.05; ∗*p* < 0.05; ∗∗*p* < 0.01; ∗∗∗*p* < 0.001; ∗∗∗∗*p* < 0.0001.

We have previously reported that LIG1 cannot discriminate against a single ribonucleotide at the 3′-end and can ligate nicks with 3′-ribonucleotide as efficiently as with canonical substrates (38). To compare the nick sealing efficiency of LIG1 in the presence of a ribonucleotide at the 3′- or 5′-end, we performed ligation assays using nick DNA substrates containing 3′-rG:C and 5′-rG:C (Fig. 7). At the time points of 0.5 to 5 min, we observed very efficient nick sealing of 3′-rG:C by LIG1 wild-type and EE/AA mutant (Fig. 7, *A* and *B*, lanes 2–7). However, at the same time points, a diminished end joining by both LIG1 proteins was obtained in the presence of nick DNA substrate containing 5′-rG:C (Fig. 7, *A* and *B*, lanes 9–14). We observed ∼10-fold difference in the amount of ligation products between 3′-rG:C and 5′-rG:C for the last time point (5 min) by both LIG1 proteins (Fig. 7, *C* and *D*).

**Figure 8:**
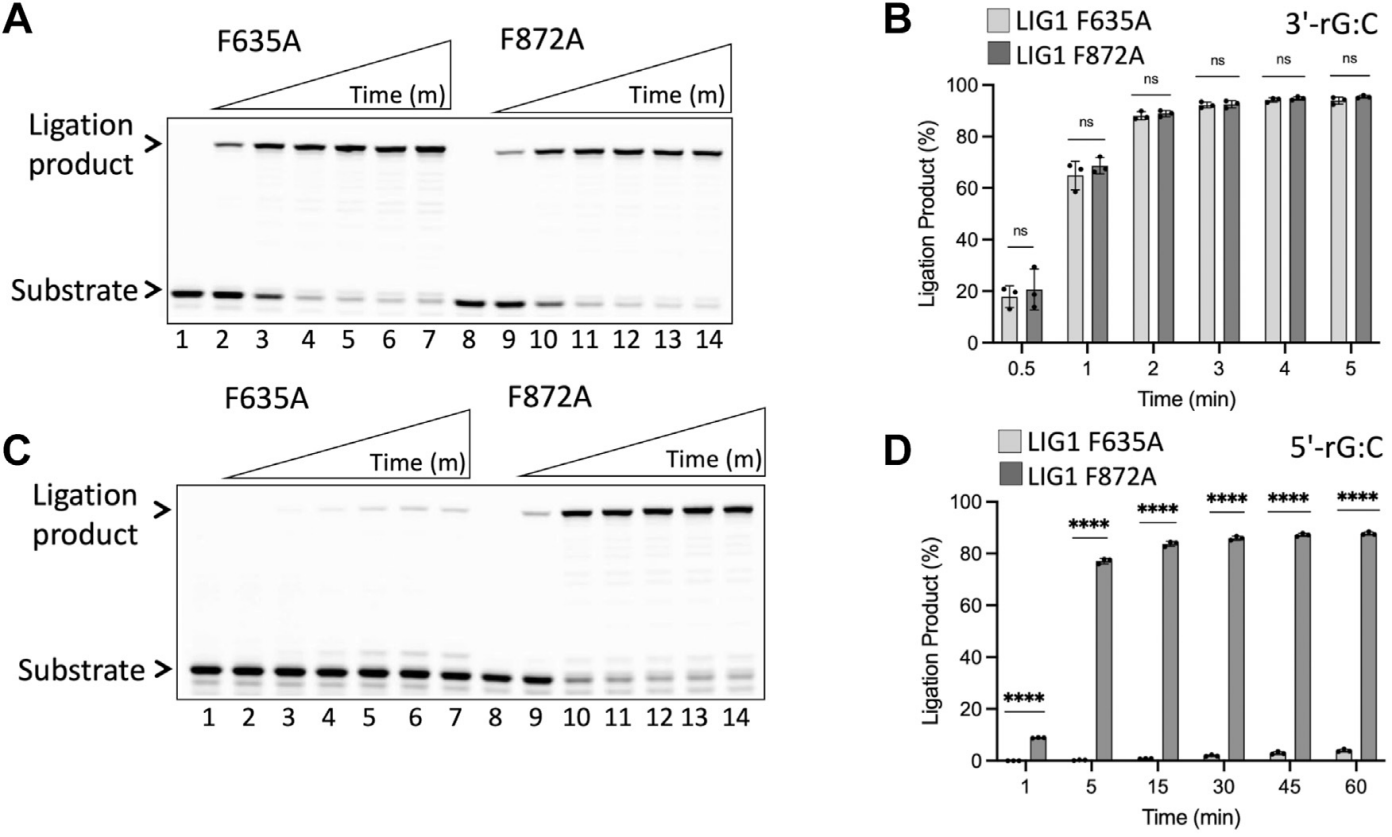
**Ligation of the nick DNA substrate with a ribonucleotide at the 3′- or 5′-end by LIG1 active site mutants.***A*, Lanes 1 and 8 are the negative enzyme controls of the nick DNA substrate with 3′-rG:C. Lanes 2 to 7 and 9 to 14 are the ligation products by LIG1 active site mutants F635A and F872A, respectively, and correspond to time points of 0.5, 1, 2, 3, 4, and 5 min. *C*, lanes 1 and 8 are the negative enzyme controls of the nick DNA substrate with 5′-rG:C. Lanes 2 to 7 and 9 to 14 are the ligation products by LIG1 active site mutants F635A and F872A, respectively, and correspond to time points of 1, 5, 15, 30, 45, and 60 min. *B* and *D*, graphs show the time-dependent change in the amount of ligation products and the data represent the average from three independent experiments ± SD; ns *p* > 0.05; ∗*p* < 0.05; ∗∗*p* < 0.01; ∗∗∗*p* < 0.001; ∗∗∗∗*p* < 0.0001.

The first X-ray crystal structure of LIG1 has demonstrated that the phenylalanine (Phe) residues, Phe(F)635 and Phe(F)872, push against the 3′- and 5′-ends of the nick, respectively, suggesting a crucial role of these residues in aligning DNA ends for proper catalysis (41). These residues insert into the minor groove and enforce the underwinding of several base pairs upstream the nick, suggesting their importance for nick recognition (41). To understand their roles in discrimination against a ribonucleotide at the 3′- and 5′-ends of the nick, we generated Phe(F) to Ala(A) substitution at these active site residues and performed ligation assays using LIG1 F635A and F872A mutants (Fig. 8). Our results demonstrated efficient nick sealing in the presence of a 3′-ribonucleotide by both LIG1 mutants (Fig. 8*A*). Furthermore, this efficient ligation of the nick substrate with 3′-rG:C by F635A and F872A was found to be slightly less than that of LIG1 wild-type, particularly at early time points of ligation reaction (Fig. 8*B*). However, in the presence of a 5′-ribonucleotide, we observed diminished nick sealing by LIG1 F635A mutant in the presence of 5′-rG:C (Fig. 8*C*, lanes 2–7), whereas efficient ligation by LIG1 F872A was obtained (Fig. 8*C*, lanes 9–14), demonstrating ∼80-fold difference between the mutants at the last time point (60 min) of ligation reaction (Fig. 8*D*). Furthermore, when compared to the activity of LIG1 wild-type, we observed a significant difference in the effect of F872A mutation in the presence of 5′-rG:C (Fig. S9). In the control experiment, we showed efficient ligation of nick substrate with canonical end (3′-dG:C) by F635A and F872A mutants (Fig. S6).

**Figure 9:**
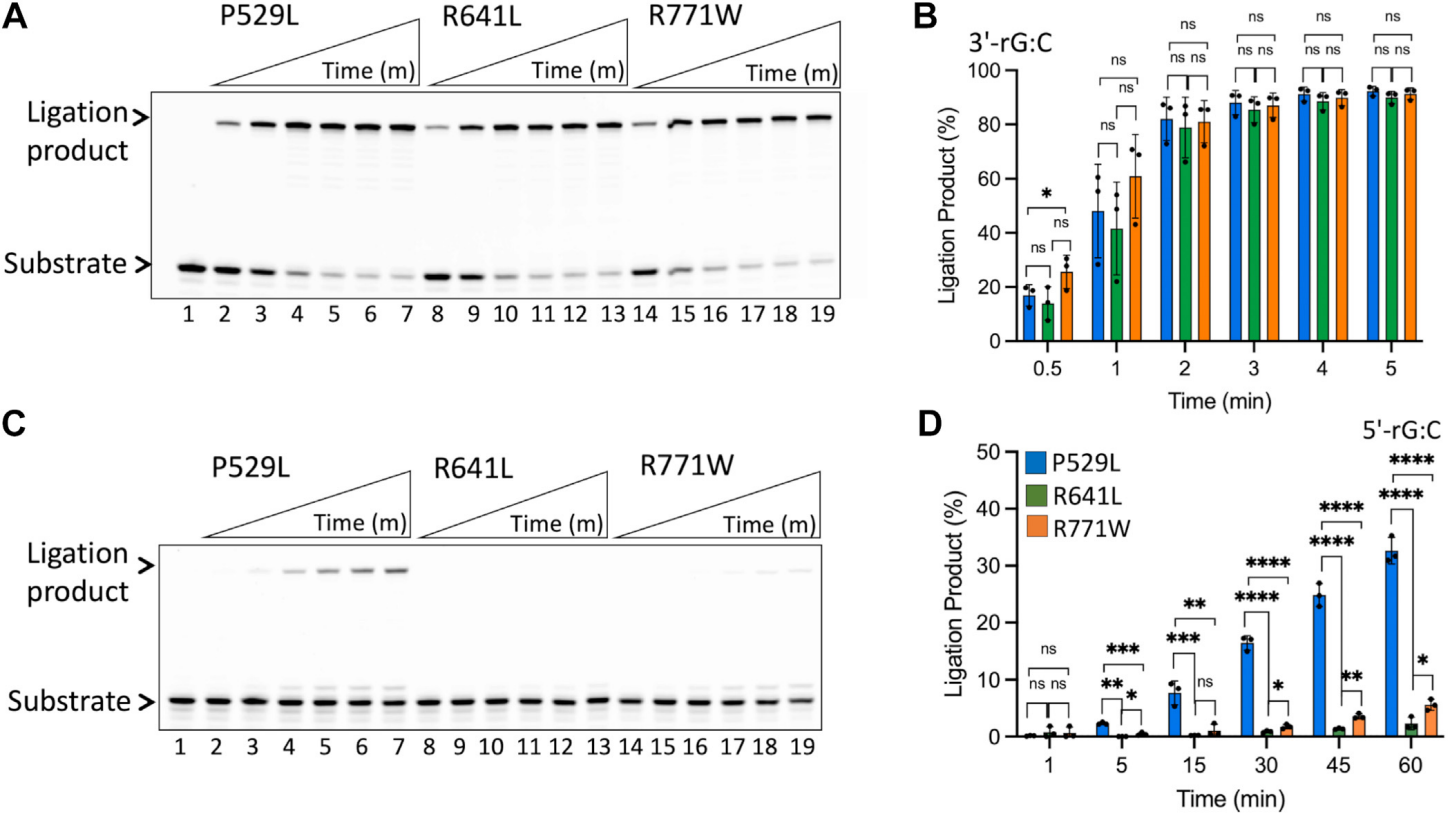
**Ligation of the nick DNA substrate with a ribonucleotide at the 3′- or 5′-end by LIG1 deficiency disease variants.***A*, line 1 is the negative enzyme control of the nick DNA substrate with 3′-rG:C. Lanes 2 to 7, 8 to 13, and 14 to 19 are the ligation products by LIG1 variants P529L, R641L, and R771W, respectively, and correspond to time points of 0.5, 1, 2, 3, 4, and 5 min. *C*, line 1 is the negative enzyme control of the nick DNA substrate with 5′-rG:C. Lanes 2 to 7, 8 to 13, and 14 to 19 are the ligation products by LIG1 variants P529L, R641L, and R771W, respectively, and correspond to time points of 1, 5, 15, 30, 45, and 60 min. *B* and *D*, Graphs show the time-dependent change in the amount of ligation products and the data represent the average from three independent experiments ± SD; ns *p* > 0.05; ∗*p* < 0.05; ∗∗*p* < 0.01; ∗∗∗*p* < 0.001; ∗∗∗∗*p* < 0.0001.

Biallelic mutations in *LIG1* located on chromosome 19 have been associated with LIG1 deficiency disease and patients exhibit hypogammaglobulinemia, lymphopenia, and immunodeficiency of variable severity (42). Cell lines carrying patient-driven mutations are susceptible to DNA damage caused by both ionizing and UV irradiation (43, 44). We have previously reported that LIG1 R641L and R771W variants show impaired catalytic activities and reduced ligation efficiencies of nick DNA containing 3′-8oxoG (45). In the present study, we compared the impact of LIG1 deficiency disease-associated mutations on the efficiency of sugar discrimination at the nick (Fig. 9). In the presence of nick DNA substrate with 3′-rG:C, efficient ligation by all three LIG1 variants was observed (Fig. 9*A*) with no significant differences in the amount of ligation products by LIG1 P529L, R641L, and R771W (Fig. 9*B*). When compared to the ligation efficiency of the wild-type enzyme, we observed relatively less efficient ligation of 3′-rG:C by LIG1 variants at early time points (Fig. S10*A*). However, in the presence of nick substrate with 5′-rG:C, although some nick sealing product by LIG1 P529L variant was observed (Fig. 9*C*, lanes 2–7), the ligation efficiencies of R641L and R771W were completely diminished (Fig. 9*C*, lanes 8–19). When compared to the ligation products by LIG1 wild-type, we obtained significant differences with R641L and R771W (Fig. S10*B*). Similarly, LIG1 P529L variant showed a ligation profile that was found to be identical to that of wild-type enzyme for nick with canonical end 3′-dG:C, while we observed relatively less amounts of ligation product by LIG1 R641L and R771W variants (Fig. S11).

### Nick sealing of DNA–RNA junction containing a 5′-ribonucleotide leads to abortive ligation by DNA ligase 3α during RER

**Figure 10:**
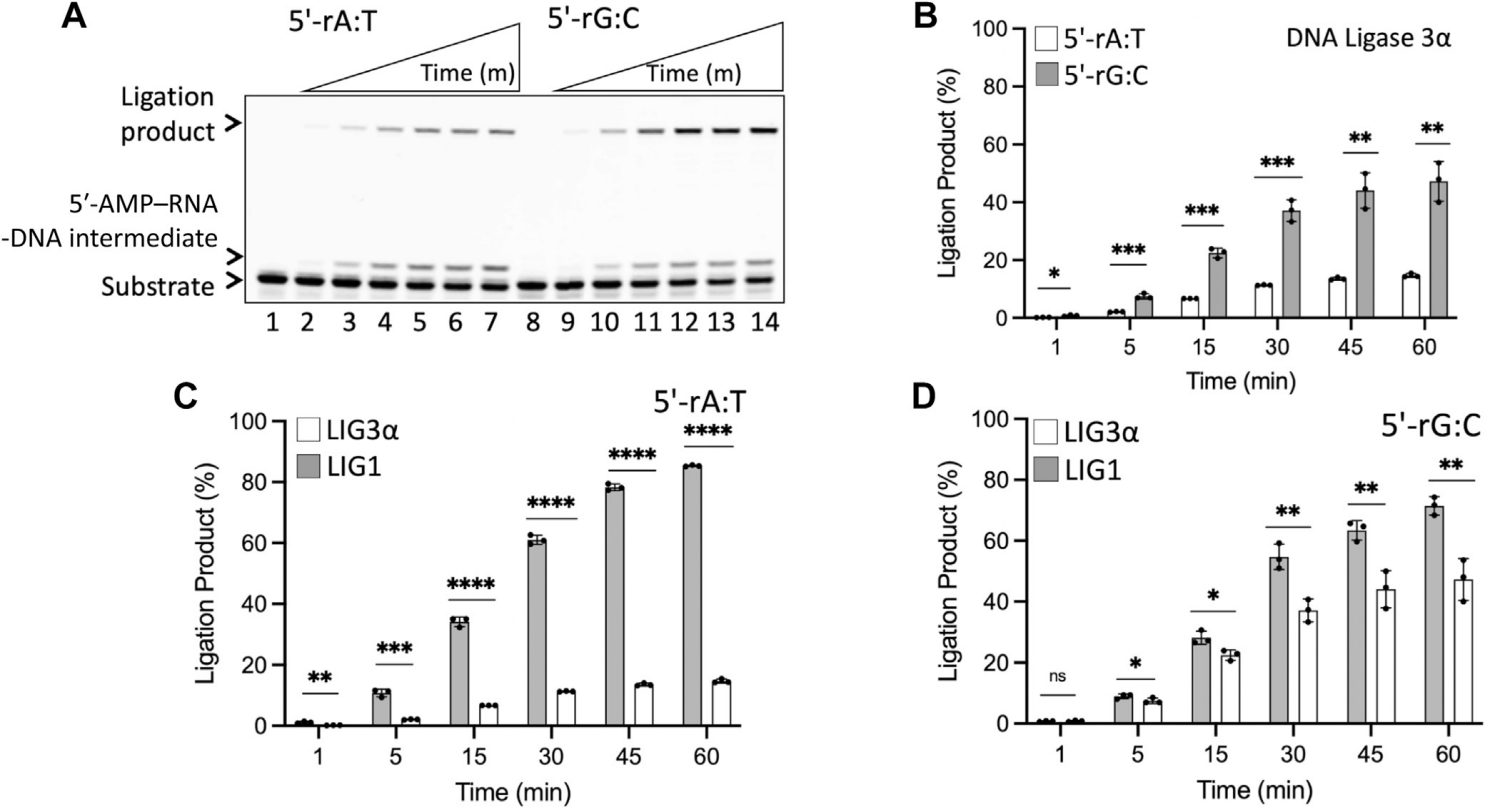
**Ligation of the nick DNA substrate with a ribonucleotide at the 5′-end by LIG3α.***A*, lanes 1 and 8 are the negative enzyme controls of the nick DNA substrates with 5′-rA:T and 5′-rG:C, respectively. Lanes 2 to 7 and 9 to 14 are the ligation products by LIG3α in the presence of 5′-rA:T and 5′-rG:C, respectively, and correspond to time points of 1, 5, 15, 30, 45, and 60 min. *B*–*D*, graphs show the time-dependent change in the amount of ligation products and the data represent the average from three independent experiments ± SD; ns *p* > 0.05; ∗*p* < 0.05; ∗∗*p* < 0.01; ∗∗∗*p* < 0.001; ∗∗∗∗*p* < 0.0001.

Human LIG1 and LIG3α share the highly conserved catalytic core and display distinct fidelity profiles against non-canonical nicks containing mismatches or damaged bases at the 3′-end (46, 47). To further investigate the efficiency of sugar discrimination by human DNA ligases against 5′-RNA–DNA junctions, we compared the ligation efficiency of nick DNA substrates containing 5′-rA:T and 5′-rG:C by LIG3α (Fig. 10). The results showed nick sealing of both substrates by LIG3α (Fig. 10*A*), and we observed a slightly high amount of ligation products in the presence of 5′-rG:C (Fig. 10*B*). However, the ligation efficiency of nick with 5′-rA:T was relatively higher by LIG1 (Fig. 10*C*) and was similar for 5′-rG:C (Fig. 10*D*). Notably, we obtained ligation failure products with 5′-AMP-RNA-DNA by LIG3α in the presence of 5′-rA:T and 5′-rG:C, which was significantly higher than that of LIG1 (Fig. S3). The end joining ability of LIG3α was found to be relatively less in the presence of nick DNA substrate with 3′-dG:C end (Fig. S4).

APTX resolves abortive ligation products upon the ligase failure on non-canonical nicks by catalyzing the release of an adenylate (AMP) moiety covalently linked to 5′-phosphate termini at the nick (31). Because of the formation of ligation failure products when LIG1 and LIG3α attempt to seal the RNA–DNA junctions with a 5′-ribonucleotide (Figs. 2 and 10), we also tested the function of APTX on these RER intermediates. Consistent with the previous report (30), we observed the removal of 5′-AMP from the nick substrate containing 5′-AMP-RNA-DNA junction, which was less efficient than that of the regular nick substrate of APTX with 5′-AMP–DNA (Fig. S12*A*). Our results showed a relatively high amount of 5′-AMP removal products from the 5′-AMP-RNA-DNA substrate at long time points of reaction (Fig. S1*B*).

## Discussion

ATP-dependent DNA ligases catalyze a consecutive three-step ligation reaction including initial adenylation (ligase-AMP), a transfer of AMP to 5′-PO_4_ end of the nick (DNA–AMP), and a final phosphodiester bond formation. Human DNA ligases, LIG1, LIG3α, and LIGIV, share a conserved catalytic core that encircles a nick with each domain contacting DNA in a compact ring structure with interactions between the N-terminal DBD and the C-terminal OBD (46). LIG1 ligates over 10 million Okazaki fragments during each round of DNA replication and catalyzes nick sealing at the final step of DNA repair pathways (35). In addition, LIG1 seals a nick at the final step of the RER pathway initiated by the cleavage of DNA at the 5′-side of ribonucleotide by RNase H2 (22). This initial processing of rNMP generates a 3′-OH end that is elongated by the replicative polymerase δ or ϵ and proliferating cell nuclear antigen (PCNA) through strand displacement. During the downstream steps of RER pathway, a 5′-rNMP-terminated flap is removed by the nuclease FEN1, and the resulting nick of the intact DNA strand is finally sealed by LIG1 (25). This RER intermediate containing 3′-OH and 5′-phospho-ribonucleotide at the nick could also serve as a substrate for LIG1 upon RNase H2-mediated cleavage of embedded rNMPs at the early steps of the RER pathway. However, how LIG1 discriminates such 5′-RNA–DNA junctions and the mechanism by which the active site of the ligase surveils nick DNA containing 5′-ribonucleotide at atomic resolution remain unknown.

We have previously reported that LIG1 cannot discriminate against RNA-DNA junctions harboring a ribonucleotide at the 3′-end of the nick (38). This lesion mimics a repair intermediate with 3′-ribonucleotide and 5′-PO_4_ at the nick, which could be formed after the incorporation of rNTPs by DNA polymerases during DNA repair in the nucleus or mitochondria (48). In the present study, we solved the structures of LIG1/5′-RNA–DNA heteroduplexes in the wild-type and low-fidelity EE/AA mutant background, both of which share a similar global conformation. We showed LIG1^WT^/5′-rG:C and LIG1^EE/AA^/5′-rG:C structures during the initial step 1 of the nick sealing reaction where AMP was bound to the active site (K568) of the ligase to be adenylated before its transfer to the 5′-PO_4_ end of nick DNA in step 2. The overlay of LIG1/5′-rG:C structures with previously reported LIG1 structures in complex with nick DNA containing canonical, mismatch, and ribonucleotide at the 3′-end demonstrated the significant conformational changes in the AdD and OBD domains in the presence of a ribonucleotide at the 5′-end of nick DNA. The AdD undergoes a drastic conformational change, during which the active site residue R738 moves ∼18 to 20 Å to form a salt bridge with 5′-PO_4_ at the nick. Furthermore, we observed that the formation of salt bridge by R589 and K744 kept 5′-PO_4_ away from the 3′-OH end of the nick for proper catalysis.

Our structures demonstrated that 5′-PO_4_ anchored by LIG1 active site residues R738, K744, and R589 led to a conformational change downstream of the nick DNA, which resulted in the narrowing down of the minor groove and widening of the major groove. This reduced interactions of DNA with the OBD and AdD of LIG1. In the LIG1/5′-RNA–DNA structure, we found a different closed state owing to the conformational change of DNA with a ribonucleotide at the 5′-end. The OBD of LIG1 normally binds to the minor groove downstream the nick DNA. However, in the structure of LIG1/5′-rG:C, we observed a narrowing of the minor groove, which initialized a major global conformational change in LIG1. A similar structural rearrangement has been previously reported for the catalytic core of LIG4, which shows “open” or “closed” state where extensive interdomain interactions occur during nick sealing (49). The study reported that the OBD of LIG4 undergoes an open (ligase–AMP) to close (DNA–AMP) conformational change that enables the ligase to encircle a nick during AMP transfer (49). Also, the study reporting the cryo-EM structure of DNA-bound ligase from *Sulfolobus solfataricus* in complex with PCNA in presence of a non-ligatable nick has shown the flexibility of OBD that does not become visible in the map and adopts multiple conformations in one of the molecules (50). Further structure/function studies with LIG1 mutants carrying amino acid substitutions at the critical residues, such as R589, K744, and R738, are required to elucidate their roles in sugar discrimination at nick DNA.

In addition to LIG1/5′-RNA–DNA structures showing that ligase stays adenylated at the step 1 of the ligation reaction, our *in vitro* ligation assays demonstrated a diminished nick sealing of 5′-ribonucleotide by LIG1 during ligation when we observed relatively efficient end joining in the presence of canonical nicks. Our results showed less extent of ligation by LIG1 F635A mutant compared to that by wild-type enzyme in the presence of 5′-ribonucleotide. The first structure of LIG1 suggested the role of F635 and F872 residues for the alignment of 3′- and 5′-ends of nick DNA, respectively, for proper catalysis (41). Our previously reported structure of LIG1/5′-dG:C also showed that DNA ends were stabilized by F635 and F872 (Fig. S13). However, LIG1/5′-rG:C structures of the present study showed that the 3′- and 5′-ends of the nick were stabilized by pi–pi interaction between +1G nucleotide (3′-dG and 5′-rG) and salt bridges at 5′-PO_4_ with R738 and K744 residues. Furthermore, we observed that F635 and F872 moved further away from both ends of the nick. This movement did not alter the stabilizing role by inducing steric hindrance at the proximity of nick site. However, we suggest that the F–A mutation at F635 could provide relatively high flexibility to 3′-end that moves away from the 5′-end of the nick. This could explain the diminished ligation of 5′-rG:C and no impact on the ligation efficiency of nick DNA with 3′-ribonucleotide by LIG1 F835A mutant. In addition to these LIG1 active site mutants, in the present study, we also showed how LIG1 deficiency disease-associated mutations affected sugar discrimination against a ribonucleotide at either end of nick DNA. While no significant difference in the nick sealing efficiency of 3′-rG:C was observed, there was ∼80-fold less ligation of 5′-rG:C substrate by LIG1 R641L and R771W variants compared to that of the wild-type enzyme. The previous study has reported that these mutations destabilize a cooperative network of LIG1–DNA interactions and Mg^2+^-binding affinity, leading to inefficient ligation of the canonical nick (51). Further structure/function studies with mutations in the active site and syndrome mutants of the ligase in complex with RNA–DNA complexes are required to clearly understand their roles in sugar discrimination by LIG1 at atomic resolution.

Overall, our previous (38) and current structures of LIG1/RNA–DNA complexes uncover the sugar discrimination strategies of the ligase in the presence of a “wrong” sugar at the 3′- or 5′- end of nick DNA and contribute to understanding how LIG1 syndrome and mutations in the active site could affect the ribonucleotide selectivity during nick sealing. We comprehensively characterized the sugar selectivity of LIG1 during the final step of most DNA repair pathways and at the initial steps of the RER pathway at structural and biochemical levels (Scheme S3). Future structural studies of other human DNA ligases with DNA–RNA complexes will be aimed at revealing how the fidelity of nick sealing is ensured to maintain genome integrity.

## Experimental procedures

### Protein purifications

DNA ligase 1 (LIG1) C-terminal (△261) wild-type protein with 6x his-tag was overexpressed in Rosetta (DE3) *E. coli* cells in Terrific Broth (TB) media with kanamycin (50 μgml^−1^) and chloramphenicol (34 μgml^−1^) at 37 °C as reported previously (52, 53, 54, 55, 56). The cells were induced with 0.5 mM isopropyl β-D-thiogalactoside (IPTG) when the OD_600_ was reached to 1.0, and the overexpression was continued for overnight at 28 °C. Cells were lysed in the lysis buffer containing 50 mM Tris-HCl (pH 7.0), 500 mM NaCl, 20 mM imidazole, 10% glycerol, 1 mM PMSF, an EDTA-free protease inhibitor cocktail tablet by sonication at 4 °C. The cell lysate was pelleted at 31,000*g* for 1 h at 4 °C. Proteins were purified by a HisTrap HP column with an increasing imidazole concentration (20–300 mM) after being equilibrated in the binding buffer containing 50 mM Tris-HCl (pH 7.0), 500 mM NaCl, 20 mM imidazole, and 10% glycerol at 4 °C. The collected fractions were subsequently loaded onto a HiTrap Heparin column that was equilibrated with the binding buffer containing 50 mM Tris-HCl (pH 7.0), 50 mM NaCl, 1.0 mM EDTA, and 10% glycerol, and then eluted with a linear gradient of NaCl up to 1 M. LIG1 proteins were further purified by Superdex 200 10/300 column in the buffer containing 20 mM Tris-HCl (pH 7.0), 200 mM NaCl, and 1 mM DTT. The protein overexpression and purifications were performed similarly for LIG1 low-fidelity mutant E346A/E592A (EE/AA), the active site mutants F635A and F872A, and LIG1 deficiency disease-associated variants P529L, R641L, and R771W. All proteins concentrated, frozen in liquid nitrogen, and stored at −80 °C.

### Crystallization and structure determination

LIG1 C-terminal (△261) wild-type (LIG1^WT^) and EE/AA mutant (LIG1^EE/AA^) proteins were used for crystallization with the nick DNA substrate containing 5′-rG:C (Table S3). LIG1-nick DNA complex crystals were grown at 20 °C using the hanging drop method as previously reported (36, 37, 38). LIG1 (at 27 mgml^−1^)/DNA complex solution was prepared in 20 mM Tris-HCl (pH 7.0), 200 mM NaCl, 1 mM DTT, 1 mM EDTA and 1 mM ATP at 1.4:1 DNA:protein molar ratio, and then mixed with 1 μl reservoir solution. Crystals were harvested and submerged in cryoprotectant solution containing reservoir solution mixed with glycerol to a final concentration of 20% glycerol before being flash cooled in liquid nitrogen for data collection (HKL Research, Inc). Crystals were kept at 100 °K during X-ray diffraction data collection using the beamlines APS-22-ID and CHESS-7B2. All structures were solved by the molecular replacement method using PHASER with PDB entry 7SUM as a search model (57). Iterative rounds of model building were performed in COOT and the final models were refined with PHENIX or REFMAC5 (58, 59, 60). All structural images were drawn using PyMOL (The PyMOL Molecular Graphics System, V0.99, Schrödinger, LLC). Detailed crystallographic statistics are provided in Table 1. The crystal structures of the present study were solved in P21 space group while previously solved LIG1 structures were solved in P212121 space group. We observed two ligase molecules in the asymmetric unit where there was no reasonable electron density for the Oligonucleotide Binding Domain (OBD) for one of LIG1 molecules.

### DNA ligation assays

Nick DNA substrates with a preinserted ribonucleotide at the 5′-end were prepared to evaluate the ligation efficiency of LIG1 in the presence of 5′-rA:T and 5′-rG:C (Scheme S2) as reported previously (52, 53, 54, 55, 56). In addition, we used the nick DNA substrates with a preinserted ribonucleotide at the 3′-end (3′-rG:C) and a canonical 3′-dG:C (Table S4). The reaction containing 50 mM Tris-HCl (pH: 7.5), 100 mM KCl, 10 mM MgCl_2_, 1 mM ATP, 1 mM DTT, 100 μgml^−1^ BSA, 1% glycerol, and nick DNA substrate (500 nM) was initiated by the addition of LIG1 (100 nM). The reaction samples were incubated at 37 °C, stopped by quenching with an equal amount of the buffer containing 95% formamide, 20 mM ethylenediaminetetraacetic acid, 0.02% bromophenol blue and 0.02% xylene cyanol, and collected at the time points indicated in the figure legends. The reaction products were then separated by electrophoresis on an 18% denaturing polyacrylamide gel. The gels were scanned with a Typhoon Phosphor Imager (Amersham Typhoon RGB), and the data were analyzed using ImageQuant software. The ligation experiments were performed similarly for LIG1 low-fidelity mutant EE/AA, the active site mutants F635A, F872A, R738A, and LIG1 deficiency disease-associated variants P529L, R641L, and R771W.

## Data availability

Atomic coordinates of the reported crystal structures for LIG1 wild-type and EE/AA mutant in complex with nick DNA containing 5′-rG:C have been deposited in the RCSB Protein Data Bank under accession numbers 9BS3 and 9BS4. All relevant data are available from the authors upon reasonable request.

## Supporting information

This article contains supporting information.

## Conflict of interest

The authors declare that they have no conflicts of interest with the contents of this article.

## Supporting information

Supplemental Figures 1-13, Tables 1-4 and Schemes 1-3
