# Supplementary material for: Structures of LIG1 uncover the mechanism of sugar discrimination against 5′-RNA-DNA junctions during ribonucleotide excision repair
Source: J Biol Chem. 2024 Aug 17;300(9):107688. doi: 10.1016/j.jbc.2024.107688 (PMC11418127; doi:10.1016/j.jbc.2024.107688)
Supplement: Supplemental Figures 1-13, Tables 1-4 and Schemes 1-3 [file mmc1.pdf]

Structures of LIG1 uncover the mechanism of sugar discrimination against 5'-RNA-DNA junctions during ribonucleotide excision repair

Kanal Elamparithi Balu, Qun Tang, Danah Almohdar, Jacob Ratcliffe, Mustafa Kalaycioğlu, and Melike Çağlayan*

Department of Biochemistry and Molecular Biology, University of Florida, Gainesville, FL 32610, USA

*To whom correspondence should be addressed. Tel.: +1 352-294-8383; E-mail: caglayanm@ufl.edu

Running Title: LIG1 discriminates 5'-ribonucleotide at nick DNA during RER.

Supplementary Figures 1-13

Supplementary Tables 1-4

Supplementary Schemes 1-3

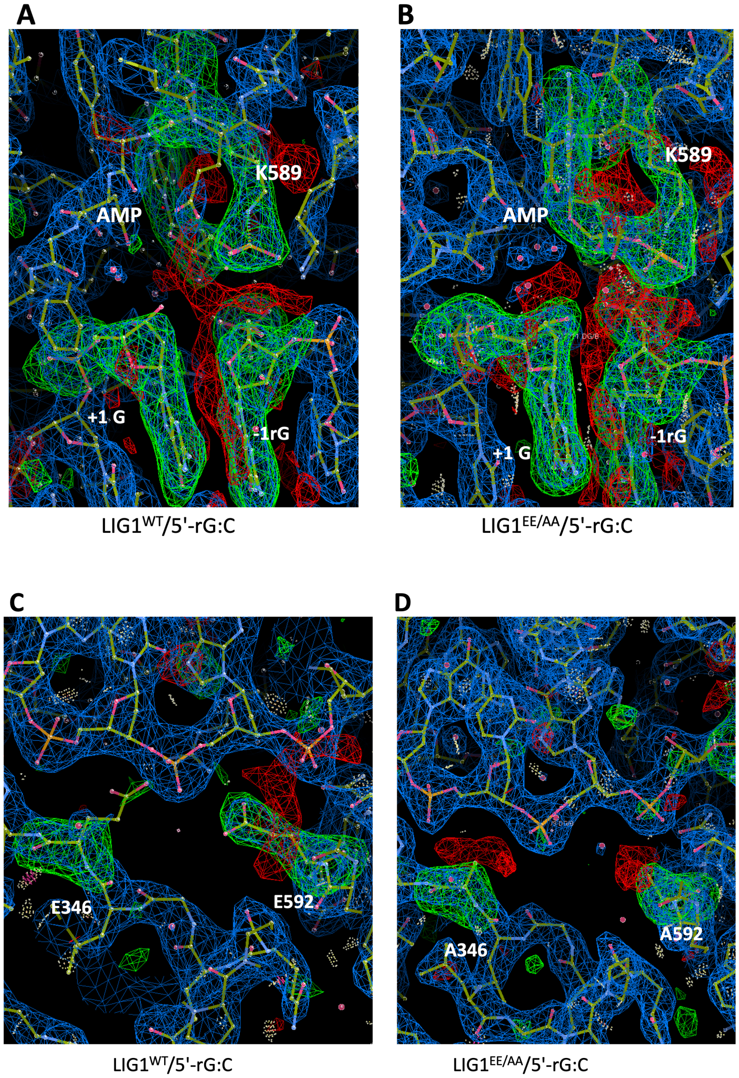

Supplementary Figure 1. LIG1/5'-RNA-DNA heteroduplex structures show the positions of AMP and the high-fidelity site (E346/A592). (A-B) Structures of LIG1 in complex with nick DNA containing 5'-rG:C are observed at the initial step 1 of the ligation reaction where AMP is bound to the ligase active site K568 residue. Simulated annealing omit maps (Fo-Fc) of the AMP are contoured at 3σ. **(C-D)** Structures of LIG1/5'-rG:C structures were solved for wild-type and EE/AA mutant that harbors E346A and E592A mutations, resulting in the ablation of the high-fidelity site (referred to as Mg^{HiFi} site).

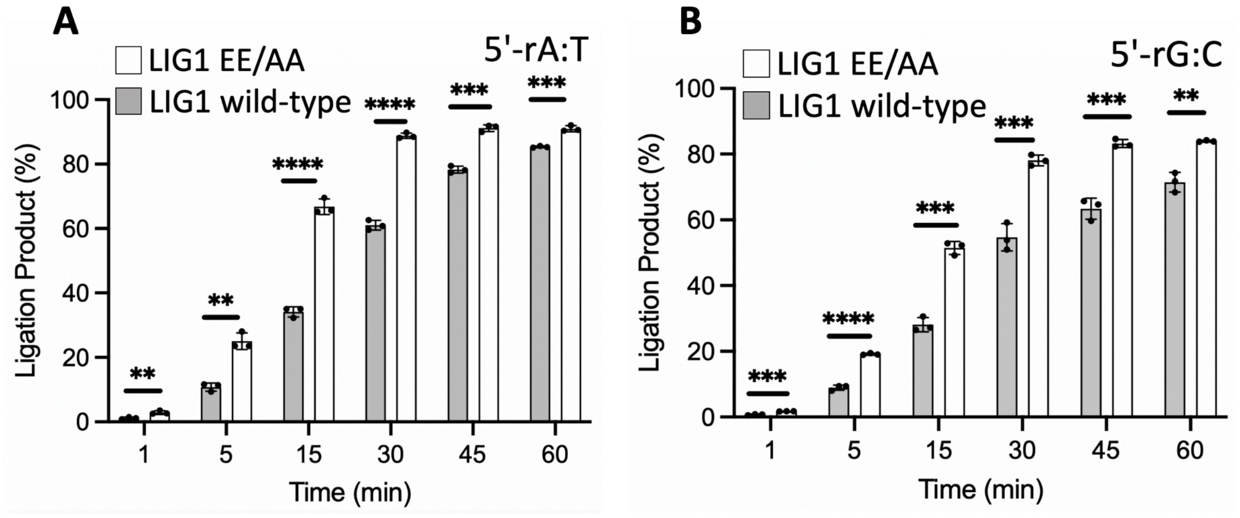

Supplementary Figure 2. The comparison of ligation products in the presence of nick DNA substrate with a 5'-ribonucleotide by LIG1 wild-type *versus* EE/AA mutant (A-B) Graphs show the time-dependent change in the amount of the ligation products in the presence of 5'-rA:T (A) and 5'-rG:C (B) by LIG1 wild-type and EE/AA mutant. The data represent the average from three independent experiments ± SD; ns $p > 0.05$; $*p < 0.05$; $**p < 0.01$; $***p < 0.001$; $****p < 0.0001$.

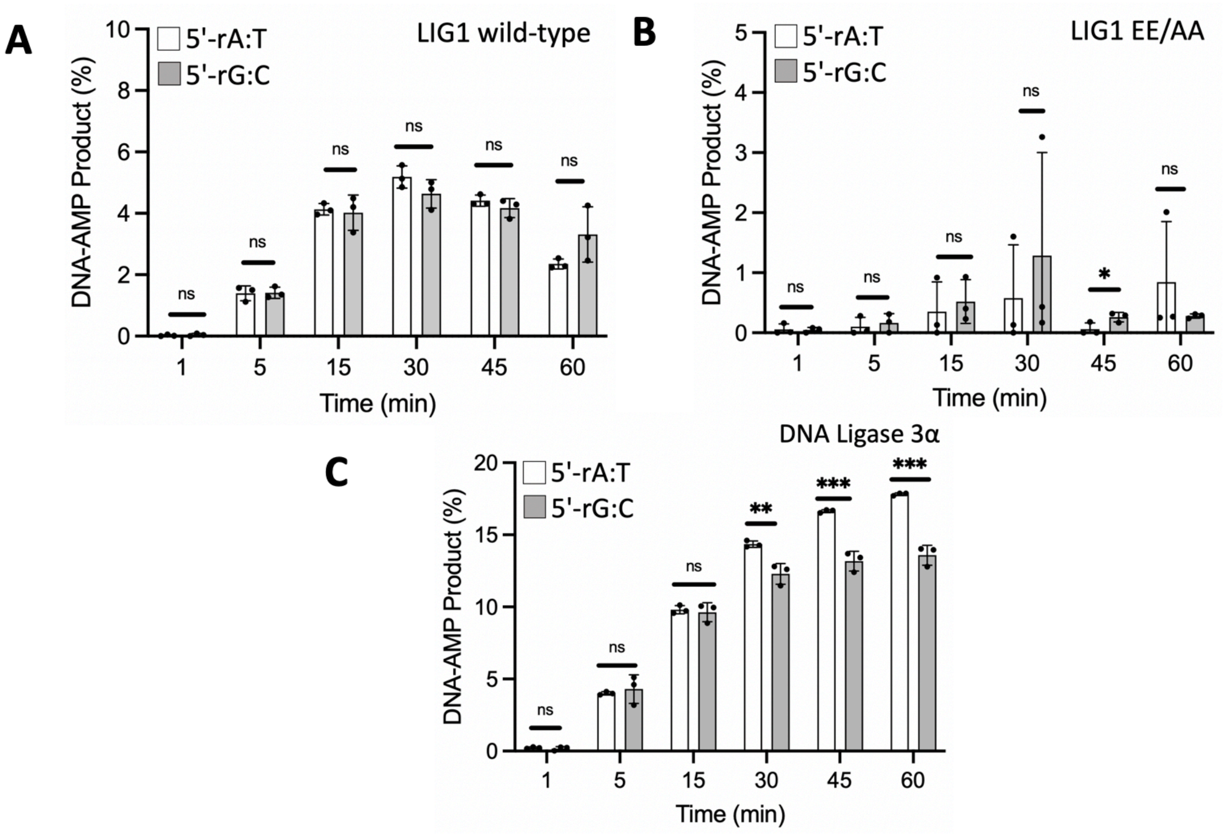

Supplementary Figure 3. The comparison of ligation failure products (DNA-AMP) in the presence of nick DNA substrate with a 5'-ribonucleotide by LIG1 and LIG3α. (A-C) Graphs show the time-dependent change in the amount of the ligation failure products with 5'-AMP-DNA in the presence of 5'-rA:T and 5'-rG:C by LIG1 wild-type (A), LIG1 EE/AA mutant (B) and LIG3α (C). The data represent the average from three independent experiments ± SD; ns $p > 0.05$; $*p < 0.05$; $**p < 0.01$; $***p < 0.001$; $****p < 0.0001$.

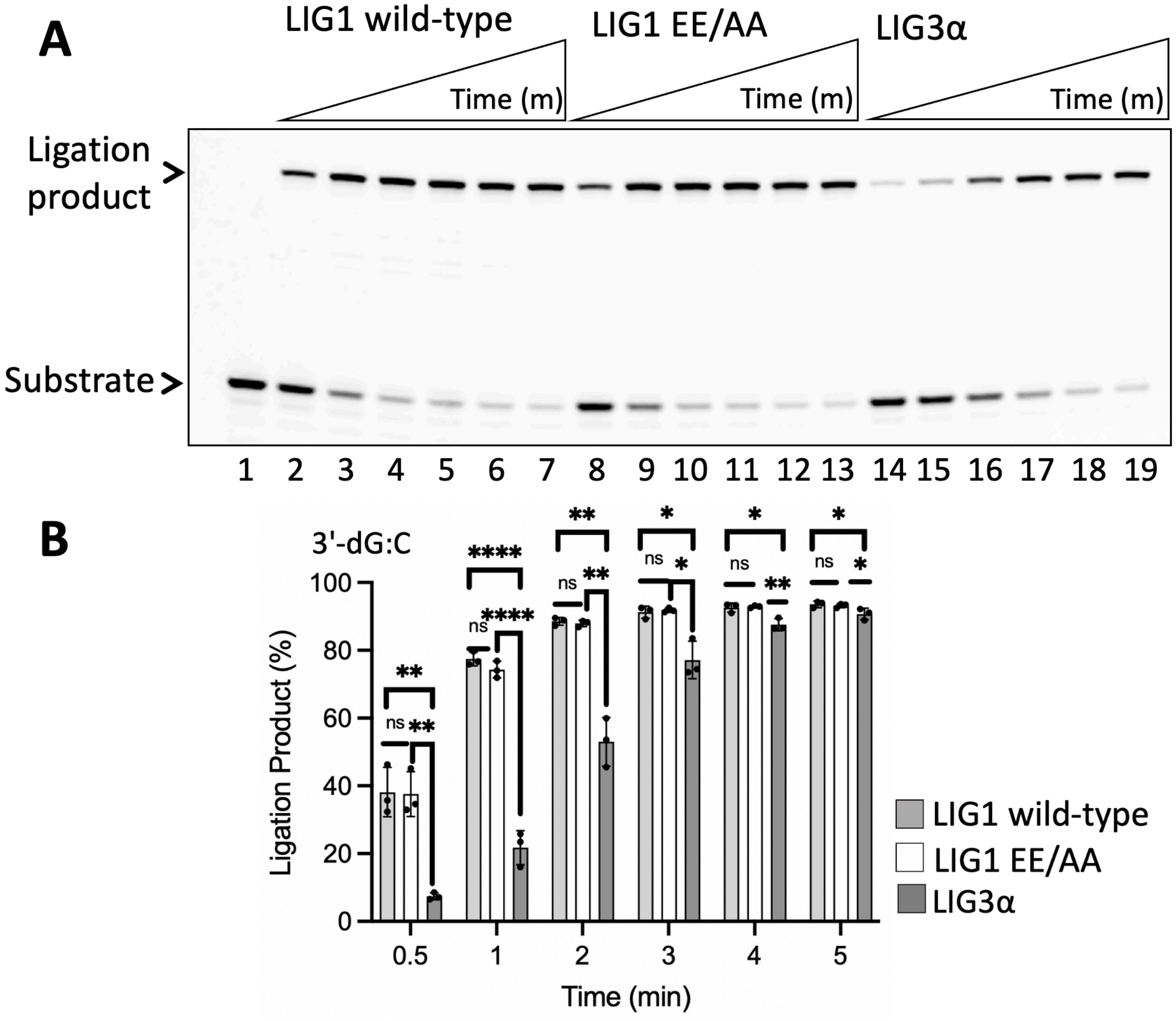

Supplementary Figure 4. Ligation of the nick DNA substrate with a canonical end by LIG1 and LIG3α. (A) Line 1 is the negative enzyme control of the nick DNA substrate with 3'-dG:C. Lanes 2-7, 8-13 and 14-19 are the ligation products by LIG1 wild-type, LIG1 EE/AA mutant, and LIG3α, respectively, and correspond to time points of 0.5, 1, 2, 3, 4, and 5 min. **(B)** Graph shows the time-dependent change in the amount of ligation products and the data represent the average from three independent experiments ± SD; ns $p > 0.05$; $*p < 0.05$; $**p < 0.01$; $***p < 0.001$; $****p < 0.0001$.

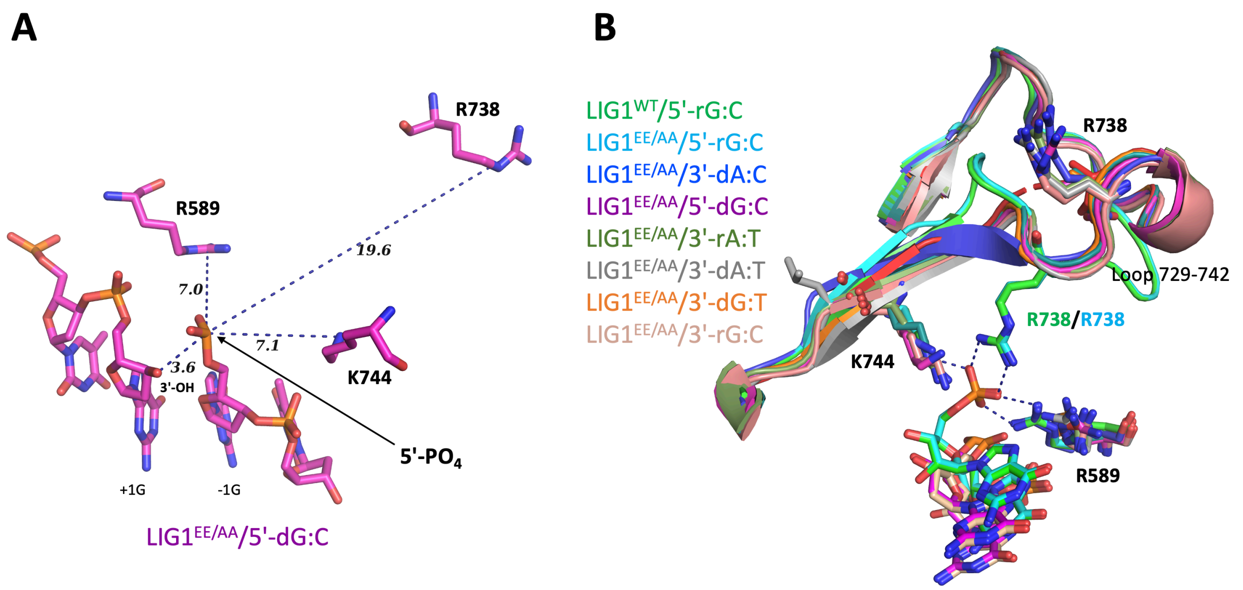

Supplementary Figure 5. LIG1/5'-rG:C structures shows a conformational change in the AdD domain of the ligase. (A) LIG1/5'-dG:C structure shows that R738 residue is away from 5'-PO_4 of the nick by ~20Å in comparison with the position of R738 in the LIG1/5'-rG:C structure as shown in Figure 4. **(B)** The overlay of LIG1/5'-rG:C structures with previously solved structures of LIG1 in complex with nick DNA containing canonical, mismatches, and 3'-ribonucleotide shows the shift in the AdD domain, particularly in the loop region corresponding to amino acids of 729-742. R738 residue in the LIG1/5'-rG:C that forms salt bridge with 5'-PO_4 and keeps the 5'-PO_4 separate from the 3'-end of the nick for proper nick sealing.

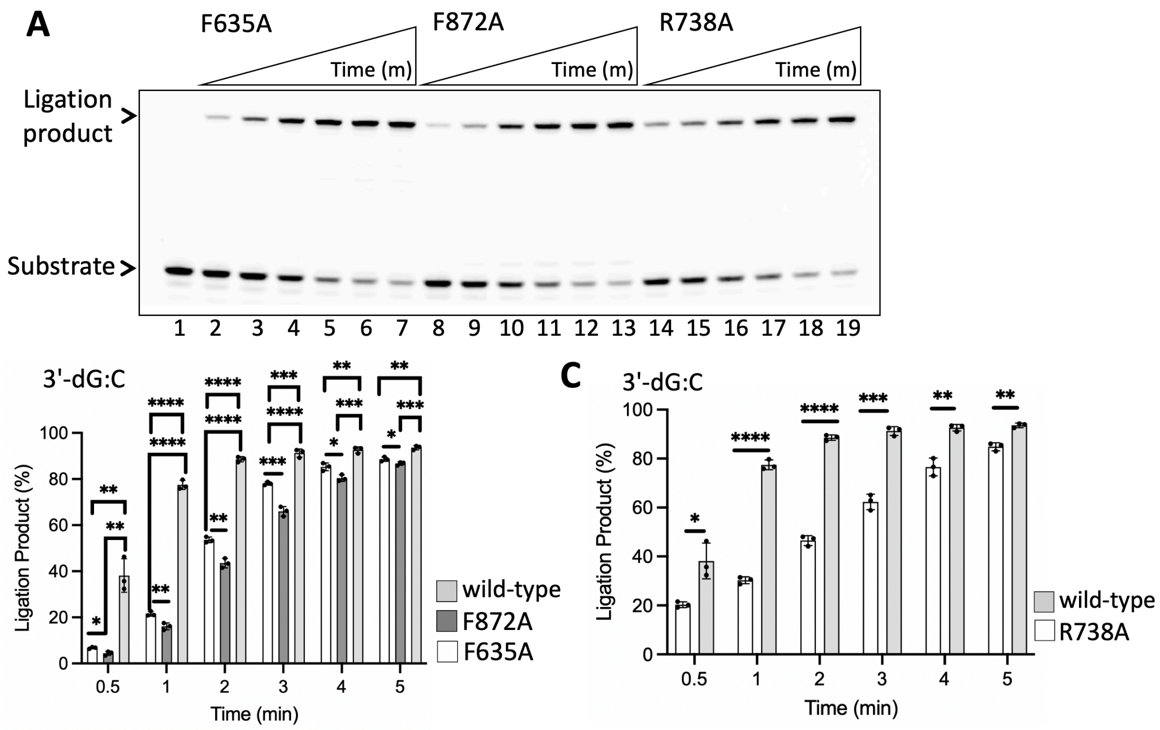

Supplementary Figure 6. Ligation of the nick DNA substrate with a canonical end by LIG1 active site mutants (A) Line 1 is the negative enzyme control of the nick DNA substrate with 3'-dG:C. Lanes 2-7, 8-13 and 14-19 are the ligation products by LIG1 active site mutants F635A, F872A, and R738A, respectively, and correspond to time points of 0.5, 1, 2, 3, 4, and 5 min. **(B-C)** Graphs show the time-dependent change in the amount of ligation products by LIG1 active site mutants and the data represent the average from three independent experiments ± SD; ns $p > 0.05$; $*p < 0.05$; $**p < 0.01$; $***p < 0.001$; $****p < 0.0001$.

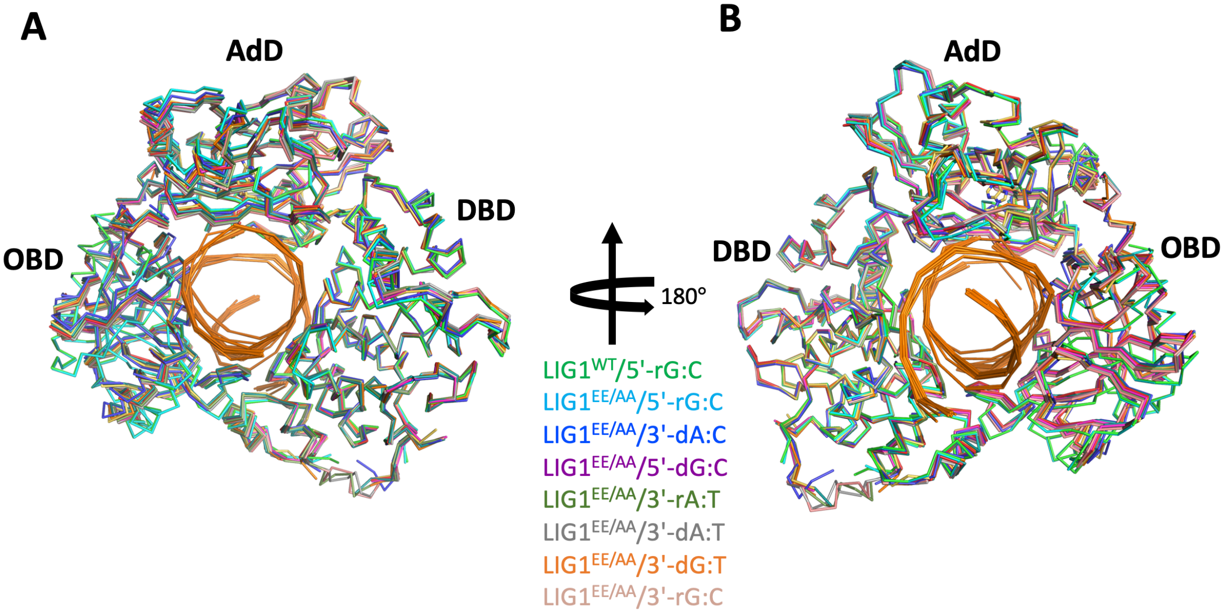

Supplementary Figure 7. The conformational change in the downstream of the nick alters the interaction of OBD at the minor groove of the DNA. (A-B) The overlay of LIG1/5'-rG:C structures with previously solved structures of LIG1 in complex with nick DNA containing canonical, mismatches, and 3'-ribonucleotide shows that the AdD and OBD domains of LIG1/5'-rG:C structures undergo conformational changes.

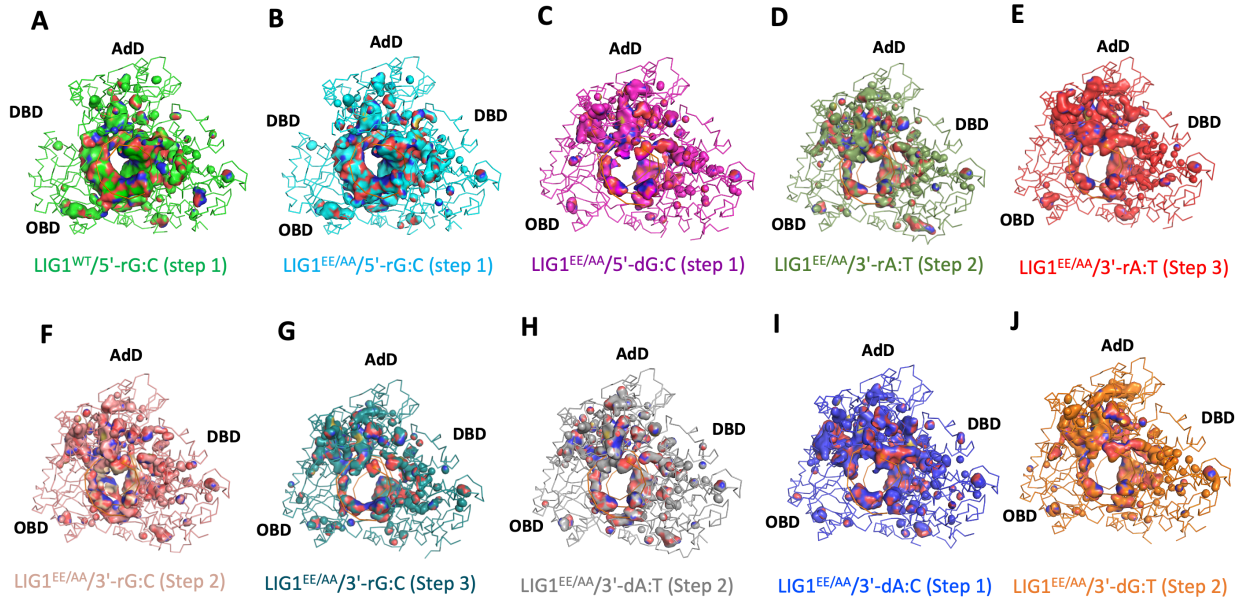

Supplementary Figure 8. Structures of LIG1/nick DNA complexes with canonical, mismatches, and ribonucleotides show the organization of the ligase catalytic core. In comparison with the LIG1/5'-rG:C structures (**A-B**), in previously solved structures of LIG1 in complex with nick DNA containing canonical, mismatches, or ribonucleotide at the 3'-end of nick (**C-J**), the conformational change in the downstream to the nick affects overall structure of DNA and reduces the interactions of AdD and OBD with the nick DNA, which increases solvent accessible area around the minor groove of the DNA. The solvent accessible area is shown in the LIG1 structures and calculated using Pymol at 3 solvent radii cutoff. The oligonucleotide binding (OBD), the adenylation (AdD), and DNA binding (DBD) domains of the ligase catalytic core are shown in individual LIG1/nick complex structures that were captured at different steps of the ligation reaction.

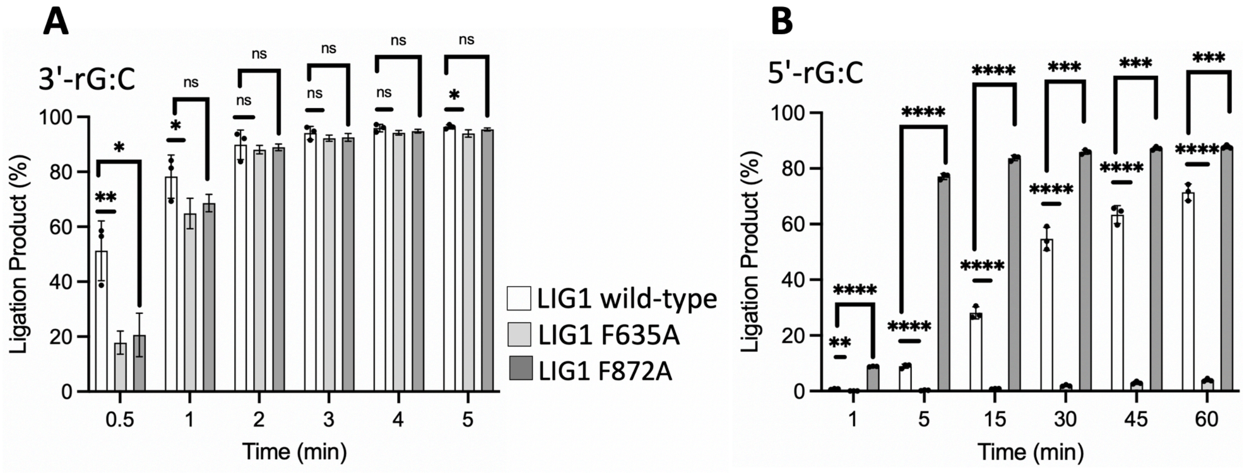

Supplementary Figure 9. The comparison of ligation products in the presence of nick DNA substrate with a 3'- *versus* 5'-ribonucleotide by LIG1 wild-type and active site mutants (A-B) Graphs show the time-dependent change in the amount of the ligation products in the presence of 3'-rG:C (A) and 5'-rG:C (B) by LIG1 wild-type and active site mutants F635A and F872A. The data represent the average from three independent experiments ± SD; ns $p > 0.05$; ∗$p < 0.05$; ∗∗$p < 0.01$; ∗∗∗$p < 0.001$; ∗∗∗∗$p < 0.0001$.

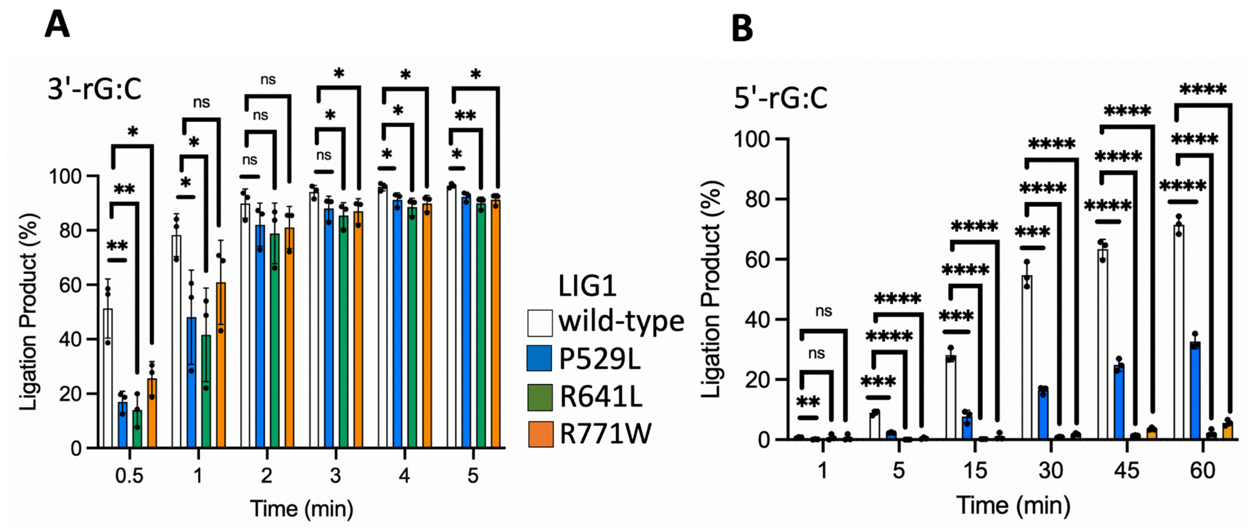

Supplementary Figure 10. The comparison of ligation products in the presence of nick DNA substrate with a 3'- *versus* 5'-ribonucleotide by LIG1 disease variants. (A-B) Graphs show the time-dependent change in the amount of the ligation products in the presence of 3'-rG:C (A) and 5'-rG:C (B) by LIG1 wild-type and variants P529L, R641L, and R771W. The data represent the average from three independent experiments ± SD; ns $p > 0.05$; $*p < 0.05$; $**p < 0.01$; $***p < 0.001$; $****p < 0.0001$.

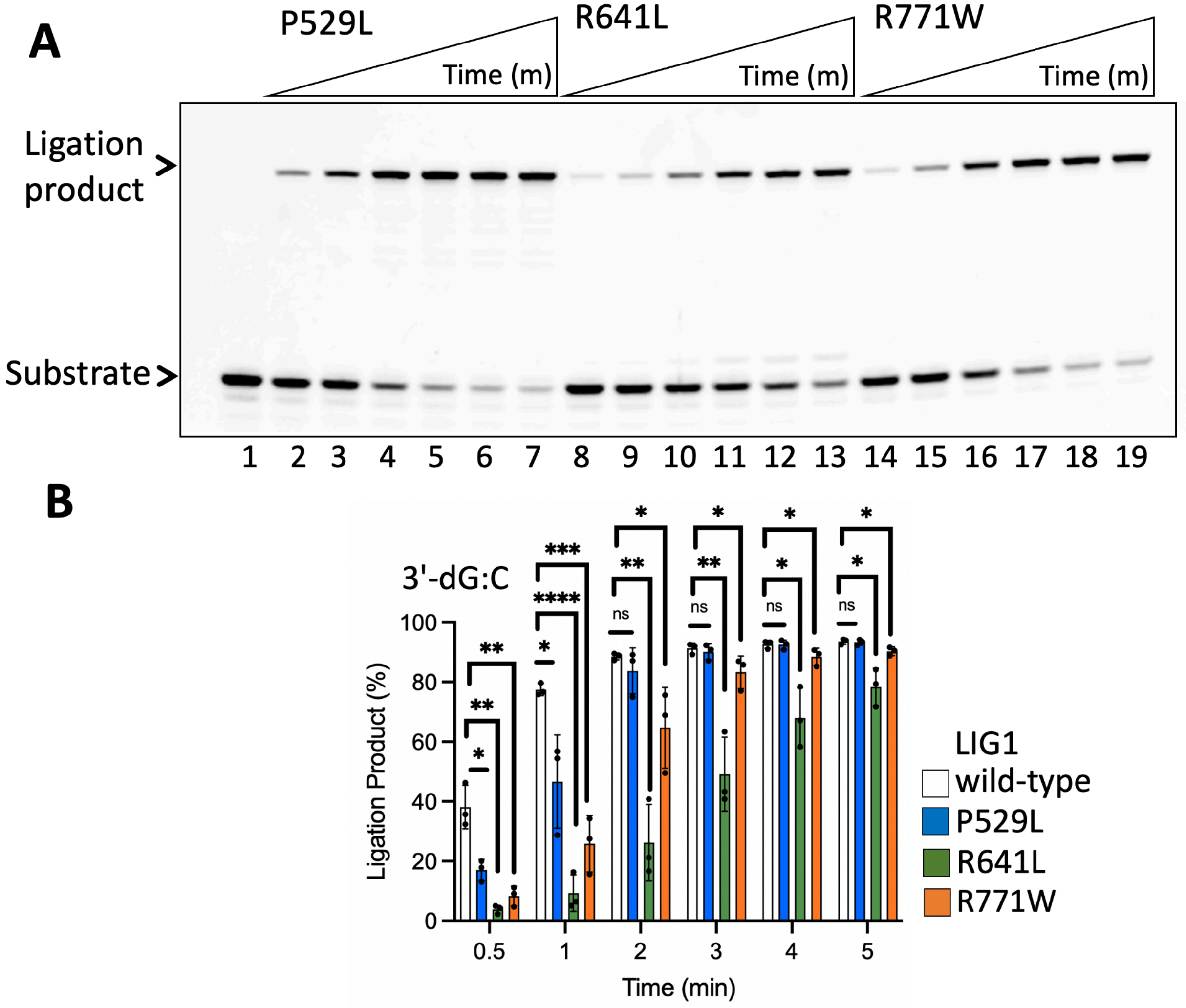

Supplementary Figure 11. Ligation of the nick DNA substrate with canonical end by LIG1 deficiency disease-associated variants. (A) Line 1 is the negative enzyme control of the nick DNA substrate with 3'-dG:C. Lanes 2-7, 8-13 and 14-19 are the ligation products by LIG1 P529L, R641L, and R771W mutants, respectively, and correspond to time points of 0.5, 1, 2, 3, 4, and 5 min. **(B)** Graph shows the time-dependent change in the amount of ligation products and the data represent the average from three independent experiments ± SD; ns $p > 0.05$; $*p < 0.05$; $**p < 0.01$; $***p < 0.001$; $****p < 0.0001$.

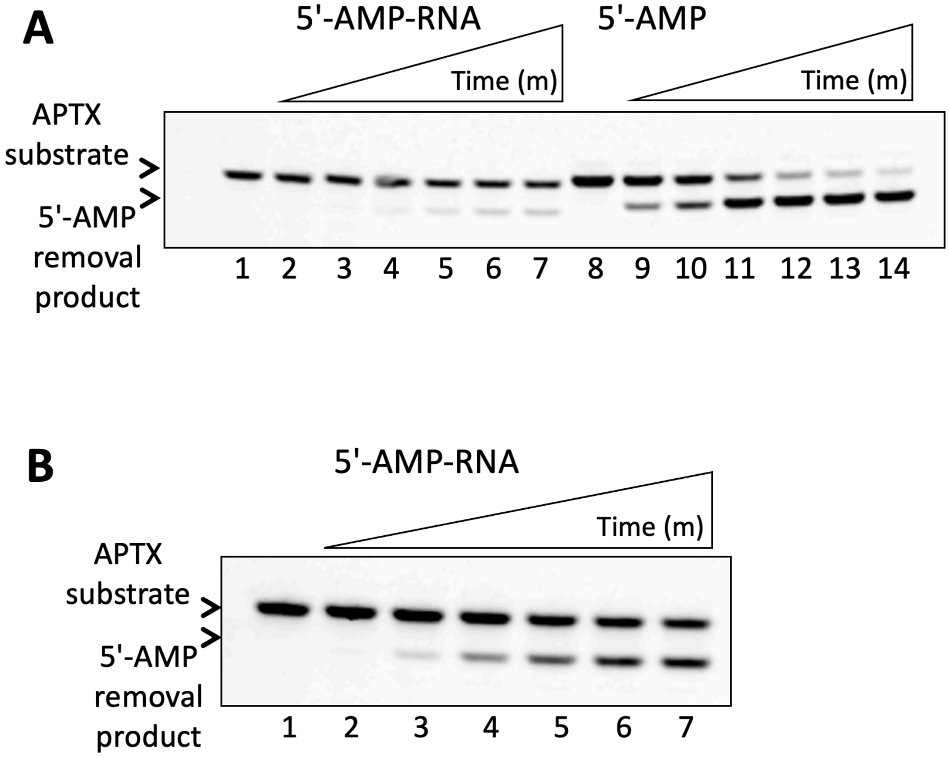

Supplementary Figure 12. Removal of 5'-AMP by Aprataxin. (A) Lanes 1 and 8 are the negative enzyme controls of the nick DNA substrates with 5'-AMP-RNA/DNA and 5'-AMP-DNA, respectively. Lanes 2-7 and 9-13 are the products of 5'-AMP removal by Aprataxin (APTX) from the nick DNA substrates with 5'-AMP-RNA/DNA and 5'-AMP-DNA, respectively, and correspond to time points of 0.5, 1, 2, 3, 4, and 5 min. **(B)** Line 1 is the negative enzyme control of the nick DNA substrate with 5'-AMP-RNA/DNA. Lanes 2-7 are the products of 5'-AMP removal by APTX from the nick DNA substrate with 5'-AMP-RNA/DNA, and correspond to time points of 1, 5, 15, 30, 45, and 60 min.

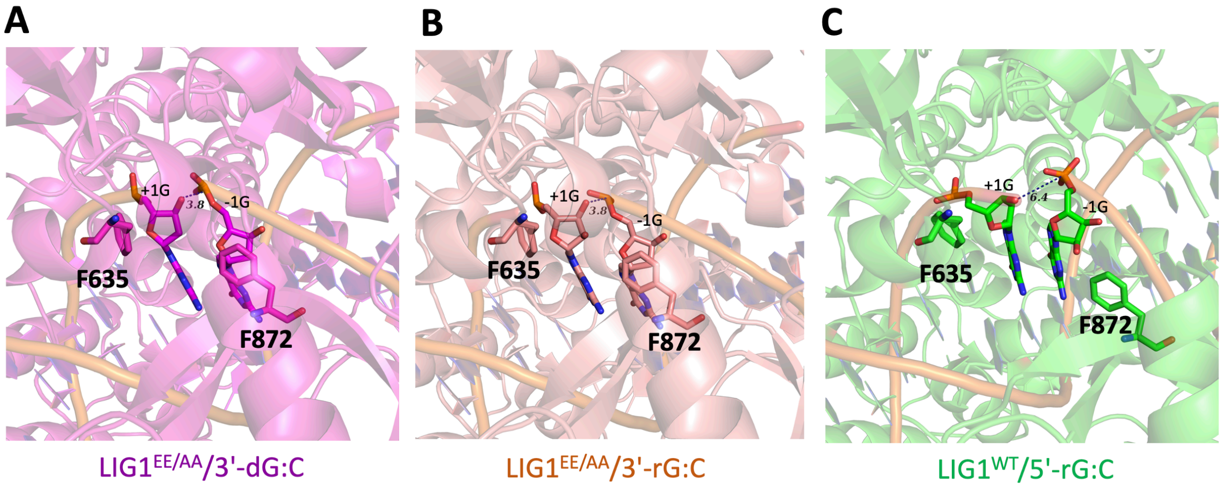

Supplementary Figure 13. Roles of LIG1 active site residues F635 and F872 for sugar discrimination. The structures of LIG1/5'-dG:C and LIG1/3'-rG:C show that 3'- and 5'- ends of nick DNA containing canonical and 3'-ribonucleotide, respectively, can be stabilized by LIG1 active site residues F635 and F872. The structure of LIG1/5'-rG:C demonstrates that 3'- and 5'-end of the nick are stabilized by pi-pi interaction between the nucleotides 3'-dG and 5'-rG and both residues F625 and F872 move away from the nick site.

**RMSD Å**	LIG1^{WT} 5'-rG:C (Step1)	LIG1$^{EE/AA}$ 5'-rG:C (Step1)	LIG1$^{EE/AA}$ 3'-dA:T (Step2)	LIG1$^{EE/AA}$ 3'-rA:T (Step2)	LIG1$^{EE/AA}$ 3'-rA:T (Step3)	LIG1$^{EE/AA}$ 3'-dG:C (Step2)	LIG1$^{EE/AA}$ 3'-rG:C (Step3)	LIG1$^{EE/AA}$ 3'-rG:C (Step3)	LIG1$^{EE/AA}$ 3'-dA:C (Step1)	LIG1$^{EE/AA}$ 3'-dG:T (Step2)
LIG1^{WT} 5'-rG:C (Step1)		0.196	1.265	1.256	0.996	1.060	1.215	1.011	0.589	0.986
LIG1$^{EE/AA}$ 5'-rG:C (Step1)			1.248	1.260	0.998	1.027	1.220	1.027	0.603	0.991
LIG1$^{EE/AA}$ 3'-dA:T (Step2)				0.236	0.537	0.393	0.444	0.444	0.970	0.539
LIG1$^{EE/AA}$ 3'-rA:T (Step 2)					0.0510	0.296	0.296	0.432	0.939	0.521
LIG1$^{EE/AA}$ 3'-rA:T (Step3)						0.298	0.471	0.296	0.668	0.379
LIG1$^{EE/AA}$ 3'-dG:C (Step2)							0.241	0.242	0.737	0.375
LIG1$^{EE/AA}$ 3'-rG:C (Step2)								0.391	0.715	0.353
LIG1$^{EE/AA}$ 3'-rG:C (Step3)									0.716	0.379
LIG1$^{EE/AA}$ 3'-dA:C (Step1)										0.655
LIG1$^{EE/AA}$ 3'-dG:T (Step2)										

Supplementary Table 1. The root mean square deviation (RMSD) of LIG1 structures solved previously and presented in this study. The RMSD values that are reported here were calculated using PyMol with the minimum of 627 atoms for 5 cycles.

DNA ligase 1	DNA	Ligation Step	PDB	Reference
LIG1$^{EE/AA}$	3'-dA:T	Step 2	7SUM	38
LIG1$^{EE/AA}$	3'-dG:T	Step 2	7SXE	38
LIG1$^{EE/AA}$	3'-dA:C	Step 1	7SX5	38
LIG1$^{EE/AA}$	3'-8oxodG:A	Step 2	9B4C	39
LIG1$^{EE/AA}$	3'-8oxodG:C	Step 2	9B4D	39
LIG1$^{EE/AA}$	3'-dG:C	Step 2	8VDN	40
LIG1$^{EE/AA}$	3'-rA:T	Pre-step 3	8VZM	40
LIG1$^{EE/AA}$	3'-rG:C	Pre-step 3	8VZL	40
LIG1$^{EE/AA}$	3'-rA:T	Post-step 3	8VDT	40
LIG1$^{EE/AA}$	3'-rG:C	Post-step 3	8VDS	40
LIG1^{WT}	5'-rG:C	Step 1	9BS3	Present study
LIG1$^{EE/AA}$	5'-rG:C	Step 1	9BS4	Present study

Supplementary Table 2. Previously solved LIG1 structures and presented in this study.

Oligonucleotide	Sequence (5'-3')
Template C	GTCCGACCACGCATCAGC
Upstream G (3'-dG)	GCTGATGCGT**G**
Downstream (5'-rG)	^{r}**G**TCGGAC

Supplementary Table 3. Oligonucleotides used in LIG1 crystallization. Upstream oligonucleotide including a canonical base at the 3'-end (dG), downstream oligo with a single ribonucleotide at the 5'-end (rG), and template oligonucleotide containing C on a template position were used to prepare the nick DNA substrate with 5'-rG:C for LIG1 crystallizations. The base at template base position is underlined and the base position at the 3'-end of nick is shown in bold.

Nick DNA Substrates	Sequence
3'-dG:C	5'-CATGGGCGGCATGAACC**G**GAGGCCCATCCTCACC-3-FAM<br>3'-GTACCCGCCGTACTTGGCCTCCGGGTAGGAGTGG-5'
3'-rG:C	5'-CATGGGCGGCATGAACC^{r}**G**GAGGCCCATCCTCACC-3-FAM<br>3'-GTACCCGCCGTACTTGGCCTCCGGGTAGGAGTGG-5'
5'-rA:T	5'-CATGGGCGGCATGAACCG^{r}**A**AGGCCCATCCTCACC-3'-FAM<br>3'-GTACCCGCCGTACTTGGCTTCCGGGTAGGAGTGG-5'
5'-rG:C	5'-CATGGGCGGCATGAACCG^{r}**G**AGGCCCATCCTCACC-3'-FAM<br>3'-GTACCCGCCGTACTTGGCCTCCGGGTAGGAGTGG-5'

Supplementary Table 4. Nick DNA substrates used in ligation assays. FAM denotes a fluorescence tag and is located at the 3'-end of the nick DNA substrates. The base at 3'-end is shown as bold and the template base is underlined.

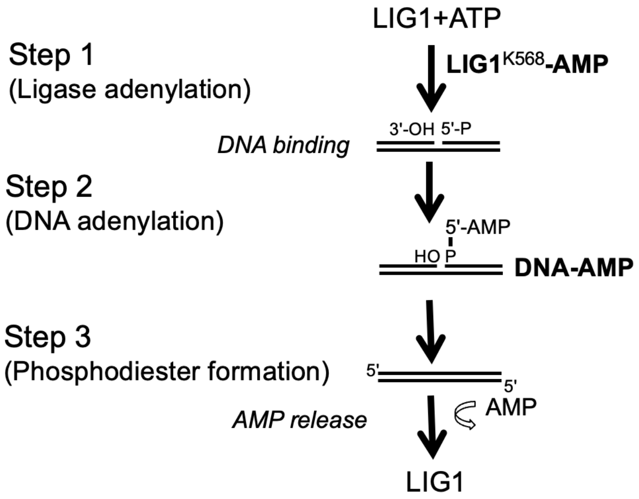

Supplementary Scheme 1. DNA ligation reaction. DNA ligation reaction includes three consequtive chemical step: the ligase attacks the α-phosphate of ATP resulting in the formation of LIG1-adenylate intermediate when AMP is linked to the ligase active site K568 residue in step 1. The ligase catalyzes the transfer of AMP to the 5'-PO_4 end of nick reulting in the formation the DNA-AMP intermediate in step 2. The ligase catalyzes in-line nucleophilic attack of the 3'-OH group onto the 5'-PO_4 and phosphodiester bond is formed coupled to AMP release in step 3.

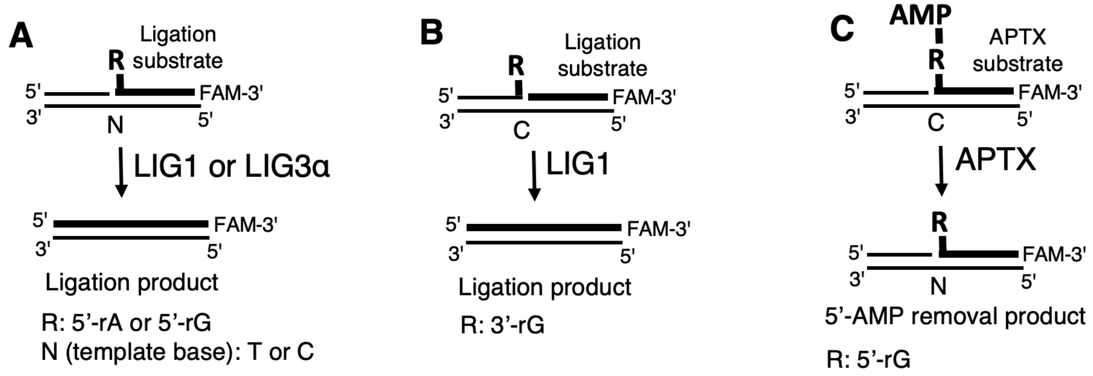

Supplementary Scheme 2. Illustration of DNA repair assays used in this study. **(A-B)** Ligation assays were used to investigate the nick sealing efficiency of LIG1 (wild-type, low-fidelity mutant EE/AA, active site mutants F635A, F872A, R738A, and LIG1 deficiency disease-associated variants P529L, R641L, R771W) and LIG3α in the presence of nick DNA substrates containing 5'-ribonucleotides (5'-rA:T and 5'-rG:C) or 3'-rG:C. **(C)** The assay was used to investigate the removal of AMP from the nick DNA substrates containing 5'-AMP or 5'-RNA-AMP by Aprataxin (APTX).

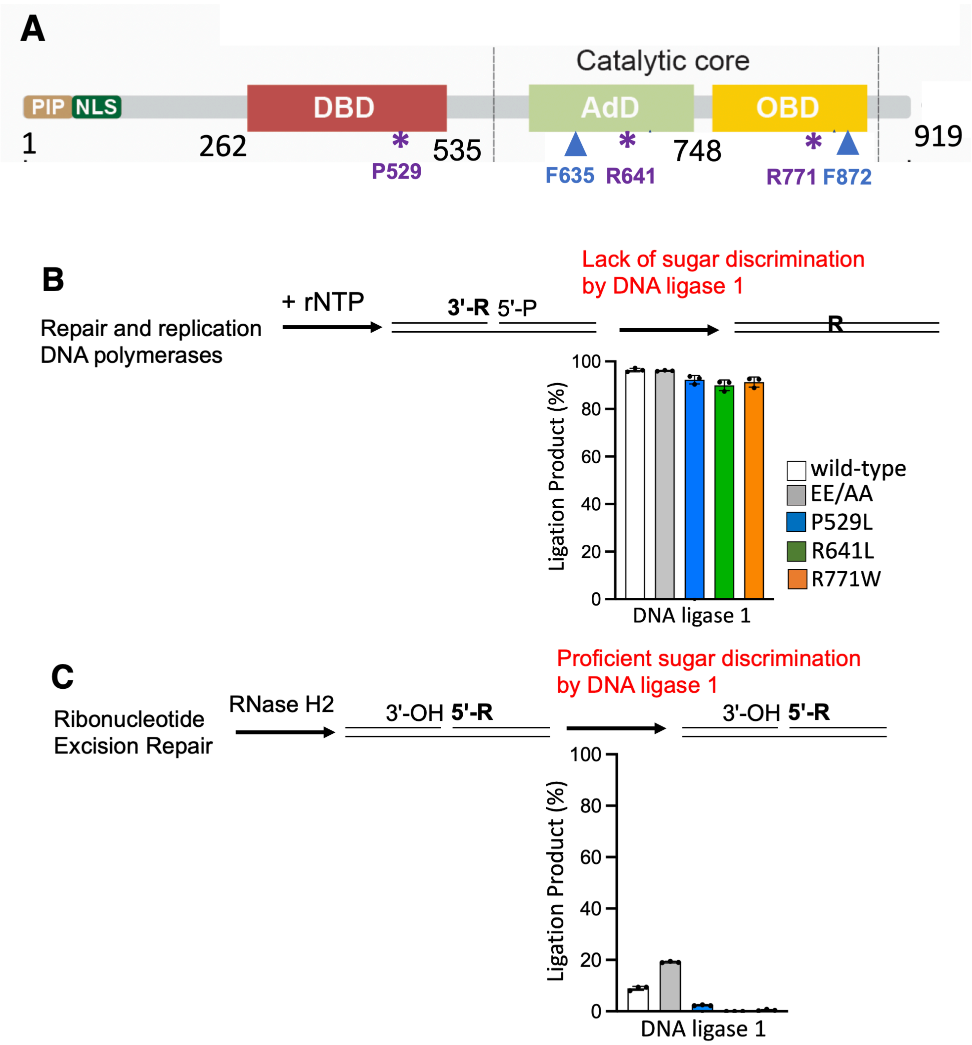

Supplementary Scheme 3. Working model of LIG1 sugar discrimination against a single ribonucleotide at the 3'- or 5'-end of nick DNA. (A) Domain organization of LIG1 protein shows the catalytic core consisting of Adenylation (AdD) and Oligonucleotide-binding (OBD) domains as well as DNA binding (DBD) domain. Unstructured and non-catalytic N-terminal region includes proliferating cell nuclear antigen (PCNA) interacting protein-box (PIP-box) and nuclear localization signal (NLS). LIG1 active site residues F635 and F872 as well as LIG1 deficiency disease-associated variants P529L, R641L, and R771W are shown based where they reside in the ligase domains. **(B)** DNA polymerases can incorporate rNTPs during replication and repair leading to the formation of RNA-DNA heteroduplex structures containing 3'-ribonucleotide (3'-R) and 5'-

PO_4 termini at nick that can be efficiently sealed by LIG1 wild-type, low-fidelity mutant EE/AA, and LIG1 deficiency disease-associated variants P529L, R641L, and R771W. **(C)** RNase H2-mediated cleavage of embedded ribonucleotides during ribonucleotide excision repair (RER) leads to the formation of RNA-DNA junctions containing 5'-ribonucleotide (5'-R) and 5'-OH termini at nick that cannot sealed by LIG1 wild-type, low-fidelity mutant EE/AA, and LIG1 deficiency disease-associated variants P529L, R641L, and R771W.
